# Tissue Engineering and Regenerative Medicine: Achievements, Future, and Sustainability in Asia

**DOI:** 10.3389/fbioe.2020.00083

**Published:** 2020-03-24

**Authors:** Fengxuan Han, Jiayuan Wang, Luguang Ding, Yuanbin Hu, Wenquan Li, Zhangqin Yuan, Qianping Guo, Caihong Zhu, Li Yu, Huan Wang, Zhongliang Zhao, Luanluan Jia, Jiaying Li, Yingkang Yu, Weidong Zhang, Genglei Chu, Song Chen, Bin Li

**Affiliations:** ^1^Department of Orthopaedic Surgery, The First Affiliated Hospital of Soochow University, Suzhou, China; ^2^Orthopaedic Institute, Soochow University, Suzhou, China; ^3^Department of Orthopaedics, Zhongda Hospital, Southeast University, Nanjing, China; ^4^Department of Otolaryngology, The Second Affiliated Hospital of Soochow University, Suzhou, China; ^5^China Orthopedic Regenerative Medicine Group (CORMed), Hangzhou, China

**Keywords:** tissue engineering, regenerative medicine, Asia, biomaterials, cell sources, biomechanics

## Abstract

Exploring innovative solutions to improve the healthcare of the aging and diseased population continues to be a global challenge. Among a number of strategies toward this goal, tissue engineering and regenerative medicine (TERM) has gradually evolved into a promising approach to meet future needs of patients. TERM has recently received increasing attention in Asia, as evidenced by the markedly increased number of researchers, publications, clinical trials, and translational products. This review aims to give a brief overview of TERM development in Asia over the last decade by highlighting some of the important advances in this field and featuring major achievements of representative research groups. The development of novel biomaterials and enabling technologies, identification of new cell sources, and applications of TERM in various tissues are briefly introduced. Finally, the achievement of TERM in Asia, including important publications, representative discoveries, clinical trials, and examples of commercial products will be introduced. Discussion on current limitations and future directions in this hot topic will also be provided.

## Introduction

Fully repairing or regenerating damaged tissues or organs and restoring their functions have been a dream of human beings. The advent of tissue engineering and regenerative medicine (TERM) appears to make it possible. Tissue engineering combines cells, scaffolds, and growth factors to regenerate tissues or replace damaged or diseased tissues, while regenerative medicine combines tissue engineering with other strategies, including cell-based therapy, gene therapy, and immunomodulation, to induce *in vivo* tissue/organ regeneration (Lysaght and Crager, 2009; Lindroos et al., 2011; Salgado et al., 2013; Porada et al., 2016). TERM is a multidisciplinary science and combines basic sciences such as materials science, biomechanics, cell biology, and medical sciences to realize functional tissue/organ repair or reconstruction. With the aging of world population trend intensifying, there is an increasing demand of organ replacements. TERM holds the potential to meet the future needs of patients (Frey et al., 2016).

The aim of TERM is to establish a three-dimensional (3D) cell/biomaterial complex, which has similar function as a living tissue/organ and may be used to repair or regenerate injured tissue/organ. The basic requirement for the complex is that it can support cell growth, transportation of nutrition and waste, and gas exchange. TERM usually uses the following three strategies: (1) cell/biomaterial complex system, in which cell-seeded biomaterials are implanted into the body to repair and regenerate tissues/organs; (2) cell systems, such as stem cell transplantation; and (3) biomaterial systems, which will be implanted into body and undergo the process of tissue integration.

Tissue engineering and regenerative medicine has been proposed and developed for more than 30 years. While several successful attempts in tissue regeneration have been achieved, TERM is still in its infancy and there are many fundamental questions that remain to be answered, including selection of cell sources, development of tissue-specific materials, development of specialized bioreactors, and construction of complex organs. More importantly, the processes and mechanisms of new tissue/organ formed using these tissue-engineered materials *in vivo*, similarity and difference between these processes with nature tissue/organ development and healing, and transformation and final destination of these materials continue to be the critical concerns in this dynamically developing field. Addressing these questions is the key to the effectiveness, stability, and security of the clinical application of tissue-engineered materials.

The aim of this review is to summarize recent major events in the broad discipline of TERM in Asia. In constructing this review, we retrieved a large number of literature and selected some representative works that are presented in this review. Indeed, we recognized that we are limited by our own small view and specialization, and that this might limit our ability to capture the comprehensiveness and diversity of TERM. Therefore, this review may not be comprehensive, but we will try our best to list some important advances in TERM in Asia.

The population of TERM research community has been rapidly expanding in Asia. In this review, a brief overview of recent development in the broad discipline of TERM in Asia will be summarized. To this end, we retrieved a large number of literature and selected representative achievements to showcase in this review. Due to the page limit, this review is far from being comprehensive; instead, we highlight here only a small fraction of important advances of TERM in Asia as examples.

## Achievement of Term in Asia

### Biomaterials

#### Polymer

Native polymers, such as acellular matrix, collagen, gelatin, hyaluronic acid (HA), silk, alginate, and chitosan, are well-known sources for preparing scaffold in TERM. There are many works to explore the interrelationship between the composition, structure, and functions of these native polymer-based scaffolds. Zeng’s group found that peripheral nerve-derived matrix hydrogel promoted remyelination and inhibited synapse formation (Zou et al., 2018). Peng and Wang’s groups used a neurotrophic peptide-functionalized, self-assembling nanofiber hydrogel to produce a neurotrophic microenvironment (Lu et al., 2018). Dai’s group developed a lot of collagen-based scaffolds for nerve regeneration (Chen et al., 2018c; Li X. R. et al., 2018). A collagen scaffold with microchannel and loading paclitaxel liposome-induced neuronal differentiation of neural stem cells (NSCs) through Wnt/β-catenin signaling could be used in spinal cord injury (SCI) repair (Li X. R. et al., 2018). In a recent work, a multichannel poly(propylene fumarate) scaffold modifying with collagen-binding neurotrophic factor 3 promoted neural regeneration of SCI (Chen et al., 2018c). They also found that controlled release of collagen-binding stromal cell-derived factor-1α (SDF-1α) from the scaffold promoted tendon regeneration in a rat Achilles tendon defect model (Sun J. et al., 2018). Chen’s group also attempted to prepare various porous collagen and gelatin scaffolds for application in TERM (Chen et al., 2014; Zhang Q. et al., 2014; Chen S. et al., 2015; Chen S. W. et al., 2017). They found that the pore structure and mechanical property also regulate cartilage regeneration (Chen S. et al., 2015). In another work, they also developed collagen porous scaffolds with parallel and concave microgrooves to regulate vascular endothelial cells and muscle cells (Chen S. W. et al., 2017). Esaki et al.’s (2019) group reported that transplantation of olfactory stem cells with biodegradable gelatin sponge could accelerate facial nerve regeneration after crush injury. They also fabricated human ear- and nose-shaped scaffolds used in gelatin and HA by integrating photocuring 3D printing and lyophilization techniques (Xia et al., 2018). The natural hydrogel such as gelatin methacryloyl (GelMA) could also be electrospun into 3D fibrous scaffolds (Sun et al., 2017). After GelMA fibrous scaffold implantation below the skin flap of rat, more microvascular formation is found. Zhang X. et al.’s (2019) group developed aligned all-cellulose nanofibers coated with bone morphogenetic protein-2 (BMP-2) via electrospinning. Aligned nanofibers instructed cell growth along fiber direction. Gao’s group reported that a HA hydrogel modified by laminin-derived peptide could promote neurite outgrowth of PC12 cells (Xing et al., 2017). In another work, they found that cell-responsive hydrogel could be used to develop a co-culture system of immune cells and smooth muscle cells (Yu S. et al., 2018). Goh’s group prepared different silk-based scaffolds for TERM (Shi et al., 2013; Teh et al., 2013). They prepared aligned fibrous scaffolds using silk fibroin, and found that it could enhance mechanoresponse and tenogenesis of mesenchymal stem cells (MSCs). Deng’s group found enhanced osteogenesis of bone marrow stromal cells (BMSCs) by a functionalized silk fibroin hydrogel (Yan et al., 2019). Cho’s group prepared an injectable microgel composed of arginine–glycine–aspartic acid (RGD)-conjugated alginate that encapsulated endothelial cells and growth factors to enhance vascularization (Kim et al., 2014). Inspired by mussel chemistry, Lu’s group developed many polyacrylamide-based polymers with good tissue adhesiveness that can be used in cartilage and skin repair (Han L. et al., 2018; Gan et al., 2019). Wang’s group used a photo-cross-linkable, injectable sericin hydrogel for minimally invasive repairing cartilage (Qi et al., 2018). Gu’s group found that chitosan/silk fibroin scaffold combined with allogeneic bone marrow mononuclear cells could facilitate nerve regeneration, and the scaffold can improve macrophage-constructed microenvironments (Yao et al., 2016; Zhao Y. H. et al., 2017).

Although nature polymers usually have good bioactivity, their mechanical properties are often weak. Compared with other materials, the properties of synthetic polymer could be easily tailored by changing the molecular structure and processing parameters. Li’s group prepared poly(ether carbonate urethane)urea scaffolds with tunable elasticity and topography, and these properties of scaffolds could regulate the fate of AF-derived stem cells through a YAP-dependent mechanotransduction mechanism (Liu et al., 2015; Zhu et al., 2016; Chu et al., 2019). Yeong et al. (2010) presented highly porous polycaprolactone (PCL) scaffold by sintering PCL powder for application in cardiac tissue engineering. The mechanical property of this scaffold could be controlled by changing porosity. Kim’s group developed a PCL-based scaffold with uniaxially aligned surface topography by stretching a 3D-printed scaffold (Yang G. H. et al., 2019). To modify the bioactivity of polymer, polydopamine (PDA)-assisted surface modification is an easy approach (Jia et al., 2019a). Poly(ether ether ketone) (PEEK) has good mechanical property and has been used as orthopedic implants. Many researchers aim to improve the biofunction of this material by endowing its antibacterial property and osseointegration property. Gao C. et al. (2017) developed a biocompatible and antibacterial coating for PEEK using PDA-based surface modification technology and subsequent deposition of silver nanoparticles, which permits its potential use as an orthopedic material. There are also many studies to design hydrogels with tunable mechanical property to mimic natural tissues. Some groups tried to prepare hydrogel with high mechanical strength. In a study, the hydrophobic, π–π interactions and chemical bonds between the polymer binders and substrate surfaces could improve mechanical performance (Shin et al., 2012). What’s more, the structure of protein could affect the strength of protein hydrogel (Fang et al., 2013). Liu group developed a lot of high-strength hydrogels (Dai et al., 2015; Xu et al., 2017; Gao F. et al., 2018). Among them, a high-strength poly(*N*-acryloyl glycinamide) hydrogel with additional function of reverse transfection and cell detachment (Dai et al., 2015). Recently, they also reported a 3D-printing high-strength gradient hydrogel synthesized by *N*-acryloyl glycinamide and *N*-(tris(hydroxymethyl)methyl) acrylamide for efficient repair of osteochondral defects (Gao F. et al., 2018). Guo’s group developed various tough hydrogels using non-covalent interaction (Guo et al., 2014; Zhao T. et al., 2014; Cui et al., 2015). A multi-responsive supramolecular hydrogel, which could be induced by temperature, light, and redox, was prepared by the formation of host–guest complexes between the polyethylene ethanol-polyhedral oligomeric silsesquioxane-(CD)7 polymer and azo-SS-azo dimer (Wang X. et al., 2017). In another study, Chen et al. (2018a) also prepared supramolecular hydrogels based on host–guest interaction; this hydrogel could protect myoblasts from deleterious mechanical stimulus. Recently, Wang Z. et al. (2018) fabricated robust and biocompatible hydrogels via host–guest interaction. When the hydrogels were damaged, the host and guest could recognize each other immediately to recombine the damaged area.

**FIGURE 1:**
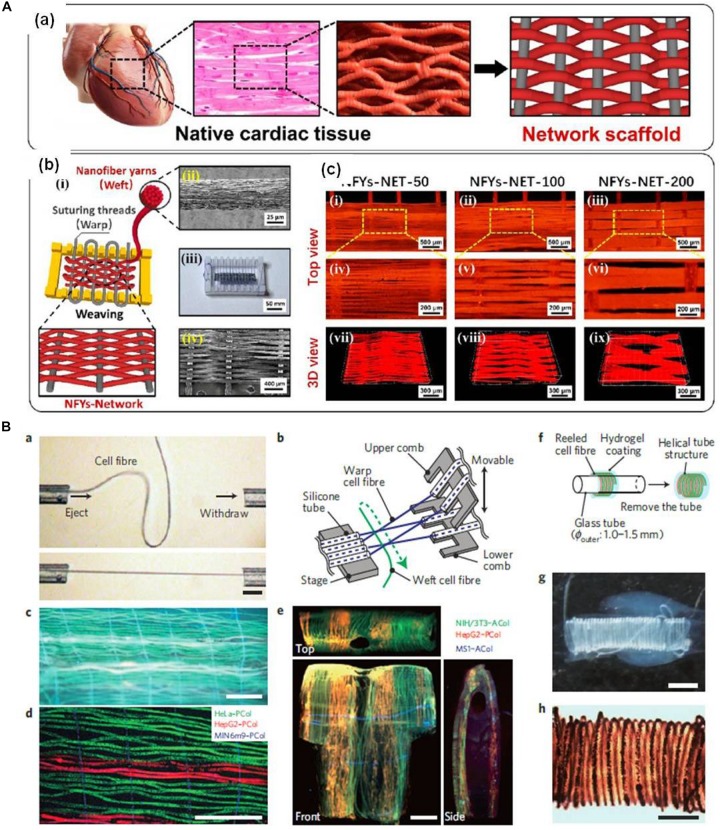
**(A)** An interwoven aligned conductive nanofiber yarn/hydrogel hybrid scaffolds for mimicking the native cardiac tissue structure (reproduced with permission from Wu et al., 2017). **(B)** A thin, long, and finely handleable cell fiber fabricated by using a microfluidic device (reproduced with permission from Onoe et al., 2013).

Up to now, many strategies were proposed for the fabrication of polymer fibers, such as electrospinning, microfluidic spinning, and 3D bioprinting. Mo’s group explores the application of electrospun fibers in TERM. They reported that modified PCL or other polymer electrospun fibers can be used in nerve (Yang G. H. et al., 2019), vascular (Liu P. X. et al., 2018), bone (Ye et al., 2019b), and other tissue regeneration in recent work. Yuan’s group also developed different electrospun fibers for drug delivery and vascular regeneration (Han et al., 2013; Zhang et al., 2013). Recently, they also encapsulated live microorganisms in electrospun fibers (Han J. P. et al., 2018) or prepared antibacterial PCL electrospun membranes containing synthetic polypeptides (Liu B. et al., 2018). Many other scholars have also done some interesting work. Yi et al. (2019) prepared aligned electrospun fibers of poly(L-lactide-co-caprolactone)/poly(L-lactic acid) (PLCL/PLLA) shell–core fibers via coaxial electrospinning and found that the stiffness of aligned fibers could regulate the phenotypic expression of vascular smooth muscle cells (VSMCs) (Yi et al., 2019). In another study, via a novel stable jet electrospinning, highly aligned hyaluronan/PLLA nanofibers with core–shell structure were also prepared for vessel regeneration (Yuan H. et al., 2016). Combining the aligned nanofibers and hydrogel, Guo’s group developed a 3D hybrid scaffold based on an aligned conductive nanofiber yarn network composed of silk fibroin, PCL, and carbon nanotubes within a hydrogel to mimic the structure of native cardiac tissue (Figure 1A; Wu et al., 2017). Microfluidic technology is also used to prepare fibrous scaffold for TERM. Matsunaga’s group prepared a gel fiber using a microfluidic device system to manufacture a phase separation polymer blend solution containing hydroxypropyl cellulose and sodium alginate (Tachizawa et al., 2017). This fiber could be used as a scaffold for *in vitro* neuronal induction. Ying’s group developed hydrodynamic spinning of gelatin-hydroxyphenylpropionic acid, alginate, poly(*N*-isopropylacrylamide), and polysulfone hydrogel fibers (Hu et al., 2010). Takeuchi’s group also described a long and finely handleable cell alginate fiber, by using a microfluidic device (Figure 1B; Onoe et al., 2013). This cell fiber could encapsulate extracellular matrix (ECM) proteins and somatic stem cells or differentiated cells and could mimic muscle fibers, nerve networks, or blood vessels *in vivo*. Gu and Zhao’s groups explored cell-laden alginate microfibers with the tunable structures through microfluidic technology (Cheng et al., 2016). Further, they demonstrated that these cell-laden fibers could be further constructed into microfiber-like tissues by stacking the microfibers. Fan’s group also co-developed a simple and reliable way to create cell-laden alginate fibers with hierarchical architecture through a microfluidic-based strategy (Wei et al., 2017). Jiang’s group constructed an artificial blood vessel with simulating autologous vascular structures from PCL and poly(lactide-*co*-glycolide) (PLGA), combining electrospinning and microfluidic technology (Cheng et al., 2017).

*In situ* forming hydrogels from polymers have also been widely used for TERM because of the ease of encapsulating proteins, drugs, genes, and cells (Yang et al., 2014; Park and Park, 2018). Various cross-linking strategies, including physical interactions (ionotropic interaction, thermo-sensitivity, and host–guest interaction) and chemical cross-linking reactions (enzyme-mediated or light-controlled cross-linking and click chemistry), have been utilized to create *in situ* forming hydrogels (Park and Park, 2018). For example, Harada’s group developed redox-responsive self-healing supramolecular hydrogel formed from host–guest polymers. A supermolecular hydrogel could quickly be formed by mixing β-CD modified poly(acrylic acid) (pAA) with ferrocene modified pAA (Nakahata et al., 2011). Photo-cross-linking hydrogels are also widely investigated. Park’s group prepared a variety of *in situ* forming hydrogels (Le Thi et al., 2017; Lee et al., 2017). An *in situ* forming gelatin hydrogel by horseradish peroxidase–tyrosinase cross-linking resulted in strong tissue adhesion (Le Thi et al., 2017). In another work, they fabricated *in situ* forming H_2_O_2_-releasing gelatin-hydroxyphenyl propionic acid hydrogels, which could be used in treatment of drug-resistant bacterial infections (Lee et al., 2017). Recently, they reported an injectable gelatin-based hydrogels that could release nitric oxide and show good antibacterial property due to the *in situ* formation of peroxynitrite (Hoang Thi et al., 2018). Hwang’s group fabricated tissue adhesive hydrogels from tyramine conjugated HA and gelatin for meniscus repair (Kim S. H. et al., 2018). This tissue adhesive hydrogel was obtained by tyrosinase-mediated cross-linking.

#### Ceramics

Being one of the important components in bone and teeth, calcium phosphate-based materials have attracted substantial attention in TERM (Wu et al., 2011). Porous calcium phosphate-based scaffolds with various compositions and controlled pore size and porosity are designed to achieve the desired biological functions. Zhao N. et al. (2017) have studied hydroxyapatite (HAp)/β-tricalcium phosphate (β-TCP) scaffolds with different weight ratios and macropore percentages, showing that scaffolds with 40% HAp and 50% macropores are optimum for cell proliferation, while 60% HAp and 30% macropores are the best for osteogenic differentiation. Another study conducted by Chen Y. et al. (2015) have revealed that porous calcium phosphate ceramics could promote angiogenic induction ability, and a higher amount of β-TCP is favorable for neovascularization of the ceramics. However, the mechanical insufficiency of calcium phosphate biomaterials limits their further applications in tissue regeneration. High-temperature sintering could strengthen their mechanical performance, but the crystallinity increases during the sintering process, which significantly reduces the degradability of the scaffold. An alternative way is to use polymers to strengthen the calcium phosphate matrix. Kang et al. (2017) applied Ca^2+^ as “ion glue” to improve the bonding between calcium phosphate minerals and organic polymers. Ma Y. et al. (2016) used PEGylated poly(glycerol sebacate) to enhance the calcium phosphate matrix, resulting in scaffold with enhanced mechanical behavior and osteogenic differentiation of BMSCs. Calcium phosphate nanoparticles with various sizes and shapes are also synthesized and applied in the area of tissue engineering (Lin K. et al., 2014). Cai et al. have demonstrated that the dimension of HAp nanoparticles has great influence on the biological activities of bone-related cells. In comparison with particles in diameters of 40 and 80 nm, 20-nm-sized HAp has shown the best stimulating effect to bone marrow MSCs (Cai et al., 2007).

Calcium silicate materials, particularly tricalcium silicate (Ca_3_SiO_5_, C_3_S), have shown great potential in bone regeneration area (Zhao et al., 2005; Lin Q. et al., 2014). Deng et al. (2018b) prepared 3D-printed bioactive ceramic scaffolds composed of Sr_5_(PO_4_)_2_SiO_4_ and showed that Sr and Si ions can stimulate cartilage regeneration by activating the hypoxia-inducible factor pathway. These ceramics are efficient to reconstruct the cartilage–bone interface, making them promising materials for the regeneration of osteochondral defect (Deng et al., 2018b). Another study has shown that Li_2_Ca_2_Si_2_O_7_ bioactive scaffold can promote the maturation of chondrocytes and stimulate the osteogenic differentiation of rabbit MSCs by continuous release of Li and Si ions, suggesting the bi-lineage bioactivities of these scaffold for osteochondral defect regeneration (Deng et al., 2018a). The effect of nanotopography surface on bone regeneration was investigated by Yang C. et al. They demonstrated that the needle-like structure can better enhance the bone regeneration *in vitro* and *in vivo* in comparison with pure tricalcium silicate scaffolds (Yang C. et al., 2017). Chang and Wu’s groups prepared porous akermanite (AKT, Ca_2_MgSi_2_O_7_) scaffold with lotus root-like structures via a modified 3D-printing strategy, and these scaffolds promote bone regeneration (Feng et al., 2017).

Silicon dioxide (SiO_2_) was also widely applied in TERM. For example, Bian’s group coated a photocleavable linker and Arg–Gly–Asp (RGD) peptide-bearing molecular cap on the surface of mesoporous silica by host–guest complexation. Remote control of calcium release and cell fate could be realized using these particles (Kang et al., 2018a, b). Bioactive glass, with a typical composition of 46.1% SiO_2_, 26.9% CaO, 24.4% Na_2_O, and 2.6% P_2_O_5_, is the first biomaterial that is able to bond to bone (Hench, 2006). The ions such as Si, Ca, and P released from the bioactive glass have the effect of stimulating osteogenesis and bone metabolism (Maeno et al., 2005). By doping with elements such as copper (Cu) (Zhao et al., 2015a), strontium (Sr) (Pan et al., 2010), zinc (Zn) (Wang H. et al., 2015), and boron (B) (Li X. et al., 2016), the promotion and stimulation effects on osteoblast activity and angiogenesis are greatly enhanced. More recently, mesoporous bioactive glass with ordered porosity and pore structures has been demonstrated, promising biomaterials for bone regeneration (Zhou et al., 2017). Zhao et al. (2015b) fabricated Sr-containing mesoporous bioactive glass scaffolds with high porosity and interconnected pores through 3D-printing technique. The mesoporous scaffolds have exhibited good ability to stimulate the proliferation and differentiation of MC3T3-E1 cells, excellent osteoinductive activity to enhance bone formation, and ability to promote new blood vessel formation (Zhao et al., 2015b). Xie et al. dispersed the mesoporous bioactive glass nanorods in bioactive glass sol to form scaffolds with hierarchical pore structures. The scaffolds are able to promote the biomineralization process and to stimulate the proliferation of rat BMSCs (Xie et al., 2019).

#### Metal

Metal, with high compressive strength, can satisfy the demand of load bearing in orthopedics such as replacement of hip. However, metallic scaffolds lack bioactivity and have problems such as degradability and possible release of toxic metallic ions. Non-biodegradable titanium (Ti) alloys, specifically Ti-6Al-4V, are widely used in orthopedics; they have superior biocompatibility and mechanical property to stainless steel, Co-based alloys, and pure Ti (Alvarez and Nakajima, 2009; Li Y. et al., 2009). However, Ti-6Al-4V contains vanadium, which is cytotoxic. Some researchers tried to develop new Ti alloys containing nontoxic elements such as niobium (Nb) and tantalum (Ta) (Alvarez and Nakajima, 2009). Due to the fact that the porous metallic scaffolds are more suitable for tissue ingrowth, a porous Ti-6Ta-4Sn alloy scaffold was fabricated with a space holder sintering method by Li Y. et al. which showed excellent biomechanical properties as an orthopedic implant (Li Y. et al., 2009). Surface modification is used to further improve the bioactivity of Ti alloys. Jang’s group fabricated an antibacterial Ag nanostructure on a Ti surface by a two-step process involving target-ion-induced plasma sputtering and Ag sputtering (Kim S. et al., 2018). Yamamoto’s group prepared carbon layer coating Ti and carbon-coated oxygen-diffused Ti for TERM (Yamamoto et al., 2011a, b). Liu’s group used graphene oxide films loaded with minocycline hydrochloride to modify the surface of Ti, to enhance osteogenic activity in the presence of bacteria (Qiu et al., 2019). They also found that the topography and elastic modulus could affect cell adhesion and spread on Ti surface (Chen L. et al., 2019). Kim and Cha’s groups assembled silica nanoparticle nanostructures on the surface of Ti to enhance *in vitro* osteogenesis and *in vivo* bone formation (Jo et al., 2017). Bioactive molecules are also used to modify the Ti and Ti alloys. Cai’s group fabricated a functional molybdenum disulfide/PDA–RGD coating on Ti implant to inhibit bacterial infection and improve osseointegration (Yuan et al., 2019a). Recently, a synergistic photothermal/photodynamic therapy (PTT/PDT) strategy aiming for remotely controlling the eradication of biofilm was developed (Yuan et al., 2019b). Huh’s group immobilized recombinant human platelet-derived growth factor-BB plus recombinant human BMP-2 on heparinized-Ti implants, and the results showed more new bone formation around implants (Lee S. H. et al., 2018). Nishimura’s group revealed that neuronal PAS domain 2 modified on the rough surface of Ti implant could facilitate osseointegration through neuroskeletal regulatory pathways (Morinaga et al., 2019). Like Ti, Ta possesses a low modulus similar with natural subchondral and cancellous bone that could facilitate load transfer and reduce stress shielding. In addition, Ta could still show unique mechanical properties even at high porosity (>80%), which allows rapid bone ingrowth (Alvarez and Nakajima, 2009). In a comparison study by Wang H. et al. (2019), porous Ta showed an equivalent biological performance with porous Ti in repairing bone. Zhang and Lei’s groups reported that Ta-coated porous Ti-6A1-4V scaffolds loading rabbit BMSCs exhibited better fusion efficacy than Ti-coated Ti-6A1-4V scaffolds in the lumbar vertebral defects of rabbits (Wang F. Q. et al., 2018). In another study by Kim’s group, the introduction of Ta on the silicone surface could reduce fibrous capsule formation (Park C. et al., 2019).

Biodegradable metals such as magnesium (Mg) and its alloys have been attracting great attention in TERM due to their satisfactory mechanical property, biodegradation property, and bioactive effect. Okuzu et al.’s (2017) group investigated the effect of Mg ions released from Mg–Ti alloy on MC3T3-E1 cell proliferation and osteogenic differentiation, and the results showed that Mg–Ti could promote early bone healing. In another study, the Mg^2+^ and Si^4+^ ions released from Mg-smectite can also promote skin regeneration (Sasaki et al., 2017). However, Mg implant has too fast degradation rate to support completely healing the tissue before its degradation. In addition, the H_2_ produced during the corrosion is also a problem. Therefore, many studies began to find ways to delay its degradation, including coating ceramic coating and Ti or using Mg alloys as an alternative. Hiromoto’s group used octacalcium phosphate and HAp coatings to control the degradation speed and to improve the biocompatibility of biodegradable Mg alloys (Hiromoto and Yamazaki, 2017). Yeung’s group prepared a Ti dioxide (TiO_2_) nano-layer on the surface of ZK60 Mg substrate through Ti and O dual plasma ion immersion implantation technique (Lin Z. et al., 2019). They also constructed a TiO_2_/Mg_2_TiO_4_ nano-layer on the surface of WE43 Mg implant. With these methods, the modified Mg or Ma alloy implants showed enhanced corrosion resistance, osteoconductivity, and antimicrobial activity (Lin Z. et al., 2019). Park and Kim’s groups coated a Zn oxide/polylactic acid (PLA) nanocomposite layer on Mg alloys; this method could not only control the degradation rate but also control the surface topography of Mg alloys substrate (Mousa et al., 2018). Moreover, this coated layer also exerted antibacterial properties. In a study, Mg was also incorporated into PLGA/TCP porous scaffold by 3D printing to promote osteogenic and angiogenic properties of the scaffold (Lai et al., 2019). Iron (Fe) exhibits high mechanical strength and slow corrosion rate, which has the potential as higher load-bearing implants. Zhang and Zheng’s groups reported that the Fe-based scaffold showed good long-term biocompatibility in both rabbit and porcine model. Its corrosion products were biosafe and could be cleared away by the macrophages. Pandey’s group found that the corrosion rate of Fe scaffold could be affected by the property of interconnected micropores (Lin et al., 2017). Sun and Wang’s groups generated engineered human ventricular heart tissue based on Fe oxide scaffolds (Yang H. et al., 2019).

#### Composite Materials

The composite materials that can keep the advantages from polymer, inorganic material, and metal are developed for TERM. Collagen and HAp are the most common organic and inorganic compounds in bone. Ma X. et al. (2016) prepared biomimetic composite hydrogel for bone repair composed of collagen and HAp; alendronate (ALN), an anti-osteoporosis drug, was also incorporated into this composite hydrogel. Chen et al.’s (2018d) group prepared biphasic calcium phosphate/collagen porous composite scaffolds and incorporated dexamethasone in this composite scaffold for bone tissue engineering. Han F. et al. (2019) prepared silk fibroin/octacalcium phosphate composite scaffold for bone tissue engineering; the addition of silk fibroin could improve the weak mechanical property and poor processability of octacalcium phosphate. Tanaka’s group found that the proliferation and alkaline phosphatase (ALP) activity of MC3T3-E1 cells was enhanced when 10 wt% Zn calcium phosphate was added to calcium phosphate cement (Horiuchi et al., 2014). The combination of organic and inorganic materials can enhance the mechanical properties. Kinsuk’s group dispersed nHA particles into an electrospun solution containing polyurethane/polydimethylsiloxane to prepare a series of composite nanofibers with different mechanical properties (Drupitha et al., 2018). Ao et al. (2017) obtained electrospun cellulose/nano-HAp nanofibers, whose Young’s modulus and tensile strength were higher than commonly used polymer fibers such as PLA. In other work, calcium silicate and Mg silicate were also doped into electrospun fibers and enhanced the mechanical properties of these fibers (Wu Z. et al., 2016; Su et al., 2017). The combination of organic and inorganic materials can also improve the bioactivity. Ruszymah’s group incorporated Zn oxide nanoparticles into chitosan–collagen porous scaffolds, and the results showed that the incorporation of Zn oxide nanoparticles could both enhance the mechanical property and biocompatibility of porous scaffold (Ullah et al., 2017). Prabhakaran and Ramakrishna’s groups also found that the incorporation of inorganic nanoparticles into polymer fiber could improve the bioactivity such as protein absorption (Esfahani et al., 2015; Zhang S. et al., 2017). Nguyen Thi’s group coated electrospun PCL membrane with gelatin–silver nanoparticles; this composite membrane showed good antibacterial ability (Tra Thanh et al., 2018). Sun’s group also incorporated Ag nanowires into polyvinyl alcohol nanofibers for their antibacterial properties (Zhang Z. J. et al., 2017). Jiang’s group coated gold nanoparticles with 6-aminopenicillanic acid, and then these particles were added into electrospun fibers of PCL/gelatin fibers against bacteria (Yang X. L. et al., 2017). Tang’s group prepared nano-TiO_2_/collagen–chitosan porous scaffold, with the addition of TiO_2_, and the mechanical properties, resistance to degradation, and antibacterial ability were all improved (Fan et al., 2016). Jayakumar’s group fabricated chitosan hydrogel/nano-ZnO composites; these nanocomposite bandages enhanced wound healing by promoting faster re-epithelialization and collagen deposition (Sudheesh Kumar et al., 2012).

### Cell Sources

Cell sources are one of three important factors in TERM. Until now, identifying and acquiring sufficient numbers of cells for application in therapeutics remain a challenge (Mao and Mooney, 2015). Various stem cells, progenitor cells, and adult tissue-derived cells are widely being investigated in TERM, among them adult tissue-derived cells are the dominant cell type used in clinic because of their ready availability and perceived safety (Fisher and Mauck, 2013).

Stem cells are still the frontline in TERM, because they have indefinite cell division potential and multiple differentiation potential (Mahla, 2016). MSCs isolated from various tissues including bone marrow, adipose tissue, blood, and amniotic fluid have potential uses in TERM (Fontaine et al., 2016; Schäfer et al., 2016; Bertheuil et al., 2019). The summary of stem cell used in TERM is shown in Table 1. Except for the above stem cells, several new stem cells also show potential application in TERM. Menstrual blood-derived stem cells (MenSCs) were discovered by Meng et al. (2007) and Cui et al. (2007) a decade ago. Since its discovery, more studies have been focused on MenSCs. The representative advantages of MenSCs compared with other six sources of MSCs are the higher proliferation rates, painless procedures, and almost no ethical issues (Khoury et al., 2014; Zhao et al., 2018). Moreover, Xiang’s group proved that MenSC-derived exosomes could inhibit hepatocyte apoptosis in D-galactosamine and lipopolysaccharide-induced FHF in mice (Chen L. et al., 2017). They also found that MenSC can treat Alzheimer’s disease and acute lung injury (Xiang et al., 2017; Zhao et al., 2018). Urinary stem cells discovered by Bharadwaj et al. (2013) also showed potential application in TERM. The urinary stem cells possess robust proliferative potential and multi-potent differentiation potential (Zhang D. et al., 2014). Recently, they reported the potential use of urinary stem cells in urinary tract reconstruction (Chen L. et al., 2017). Lee and Kwon groups reported that the neuronal differentiation of human urine-derived stem cells could be promoted by the presence of laminin and platelet-derived growth factor-BB (Kim J. Y. et al., 2018). Wang and Wu’s groups found that the exosomes secreted by urine-derived stem cells could repair pubococcygeus muscle injury and improve stress urinary incontinence in rats (Wu R. et al., 2019).

**TABLE 1:** Examples of MSC used in tissue engineering and regenerative medicine in Asia.

Cells	Disease	Mode of stem cell application	References
Bone marrow stem cell (BMSC)	Bone injury	1. BMSC and endothelial progenitor cell mixture for large segment of bone defect 2. Calcitonin-related gene-modified rat BMSCs for skull defect 3. Repair of oligo(poly(ethylene glycol)fumarate) hydrogels seeded with BMSCs for repairing osteochondral defects	Peng et al., 2019 Yu et al., 2019 Lim et al., 2013
	Cartilage injury	1. Reconstruction of human-ear-shaped cartilage by co-culturing chondrocytes with BMSCs 2. *In vitro* engineered cartilage based on autologous BMSC	Zhang L. et al., 2014 He et al., 2017
	Spinal cord injury	1. BMSC-derived extracellular vesicles to repair spinal cord injury 2. Exosomes derived from BMSCs repair traumatic spinal cord injury	Lu Y. et al., 2019 Liu W. et al., 2019
	Lung injury	1. Mice BMSCs were injected into mice via the tail vein to treat acute lung 2. BMSC transplantation to treat smoke inhalation injury	Feng et al., 2019 Liang et al., 2019
	Liver cirrhosis	Autologous BMSCs transplantation in human	Rajaram et al., 2017
	Severe Asherman syndrome	GFP-labeled BMSCs was injected systemically through the tail vein in rat	Gao L. et al., 2018
	Intrauterine adhesion	BMSC-loaded elastic poly(glycerol sebacate) scaffold	Xiao et al., 2019
	Hearing loss	BMSC transplantation recovery functional restoration of mouse cochleae	Kada et al., 2019
	Peripheral nerve injury	BMSC-derived acellular matrix-coated chitosan/silk scaffold	Xue et al., 2017
	Type 2 diabetes	Autologous BMSCs transplantation in human	Wang et al., 2011; Bhansali et al., 2014
Adipose mesenchymal stromal cell (ADSC)	Osteoarthritis	ADSCs with hyaluronic acid were intra-articularly injected into the knee	Kuroda et al., 2015
	Cartilage injury	1. ADSC/hyaluronic acid (HA)-modified thermoresponsive poly(*N*-isopropylacrylamide) hydrogel 2. Controlled chondrogenesis from ADSC by recombinant transforming growth factor-beta 3 fusion protein in peptide scaffolds	Wang C. Z. et al., 2018 Zheng et al., 2015
	Bone injury	MiR-135-modified ADSC	Xie et al., 2016
	Intervertebral disc injury	ADSC-derived tissue-engineered construct	Ishiguro et al., 2019
	Stricture after extended esophageal endoscopic submucosal dissection	ADSC-sheet transplantation	Perrod et al., 2017
	Urethral defect	Polylactid acid fibrous membrane seeded with ADSC	Wang D. J. et al., 2015
	Atrophied muscle	Autologous ADSCs transplantation	Park and Kwon, 2017
	Type 2 diabetes	Autologous ADSCs transplantation	Qi et al., 2019

Takahashi and Yamanaka’s (2006) groups reported an induced pluripotent stem (iPS) cell, which could express the marker genes of embryonic stem cells and exhibit properties similar to embryonic stem cells (Okita et al., 2007; Hamanaka et al., 2011). With this method, Zeng and Zhou’s labs first generated several iPS cell lines that could meet the most stringent criteria for pluripotent stem cells and maintained a pluripotent potential close to embryonic stem cells (Zhao et al., 2009). In another study, when the somatic cells were exposed to transcription factors (Oct3/4, Sox2, Klf4, and c-Myc), iPS cells were formed with pluripotency (Takahashi and Yamanaka, 2006). However, reactivation of the c-Myc retrovirus with this method can increase tumorigenicity and hinder its clinical applications. In their further study, they developed a modified approach for the generation of iPS cells that avoid the use of Myc retrovirus (Nakagawa et al., 2007). Other strategies to induce reprogramming with quicker action or reducing risk of tumor formation are also developed. Subsequently, iPSCs have been successfully obtained from different species, including humans, rats, and rhesus monkeys (Okita et al., 2007; Liu et al., 2008; Kang et al., 2009; Hamanaka et al., 2011).

In addition, compared with *in vitro* reprogramming, direct *in vivo* reprogramming by retroviral injection has greater efficiency, due to avoiding the steps of *in vitro* culture and transplantation (Sadahiro et al., 2015). *In vivo*, the induced efficiency is different with *in vitro*, because transcription factors, epigenetic factors, microRNAs, secreted molecules, and cellular microenvironment are all important for cell fate specification. A work done by Deng and Ding’s labs reported that appropriate chemical cocktails were needed to add into the medium of pluripotent rat cells to maintain their characteristics (Li W. et al., 2009). This work also underscores the combined importance of the fibroblast growth factor (FGF), WNT, and transforming growth factor-β (TGF-β) pathways in regulating pluripotency in different species. To improve direct reprogramming, the strategies that combine reprogramming with morphogens could potentially be adapted for inducing cells into target cells. A variety of cells, including cardiomyocytes, vascular cells, hepatocytes, neural cells, and pancreatic cells, have been obtained from both direct reprogramming and iPS cell differentiation methods (Table 2).

**TABLE 2:** Summary of direct reprogramming by lineage-specific transcription factors.

Original cells	Reprogramming factors	Induced cells
	Oct4/Sox2/Klf4/c-Myc (mouse and human)	Pluripotent stem cell
	1. Brn2/Ascl1/Myt1l (BAM) (mouse)	Excitatory neuron
	2. BAM/NeuroD1 (human)	
	BAM/Lhx3/Hb9/lsl1/Ngn2 (mouse)	Motor neuron
	Ascl1/Nurr1/Lmx1a (mouse and human)	Dopaminergic neuron
Fibroblast	Sox2 (mouse and human)	Neural stem cell
	1. Hnf4a/Foxa1 (mouse)	Hepatocyte
	2. Hnf1a/Hnf4a/Hnf6/Atf5/Prox1/Cebpa (human)	
	Erg/Gata2/Lmo2/Runx1c/Scl (mouse)	Hematopoietic progenitor cell
	Foxo1/Er71/Klf2/Tal/Lmo2 (mouse)	Endothelial cell
	Gata4/Mef2c/Tbx5 (mouse)	Cardiomyocyte
	MyoD	Skeletal myocyte
Exocrine cell	1. Neurogenin3/Pdx1/Mafa (mouse)	Pancreatic β-cell
	2. Epidermal growth factor/ciliary neurotrophic factor (mouse)	
Myeloid cell	Run1t1/Jlf/Lmo2/Prdm5/Pbx1/Zfp37	Hematopoietic stem cell

*BAM indicates Brn2, Ascl1, and Myt1l.*

### Enabling Technologies for TERM

The rapid development of TERM is inseparable from research and development of new technologies. As tissue engineering advances, new technologies, such as 3D printing and microfluidics, have attracted a lot of attention for preparing materials or scaffolds. To obtain suitable cells for TERM, cell sheet and genome engineering have also been widely explored. To construct mature tissues/organs for *in vivo* implantation, a variety of bioreactors have been developed. In addition, microfluidics and organ-on-a-chip were also effective technologies for TERM.

#### 3D Printing

One of the developments for preparing tissue-engineered constructs is bioprinting. Bioprinting is an effective method to fabricate tissue-mimicking constructs through patterning cells, biomaterials, and biomolecules (Arslan-Yildiz et al., 2016; Tasnim et al., 2018; Matai et al., 2020). The 3D bioprinting of cells, growth factors, and hydrogels is mainly based on laser (Ali et al., 2014), droplet (Cui et al., 2014), and extrusion (Iwami et al., 2010) bioprinting. Bioprinting greatly promoted the development of skin tissue engineering. Fu’s group prepared 3D-ECM through 3D bioprinting (Huang et al., 2016). The created 3D-ECM could direct cell differentiation and promote functional skin regeneration (Huang et al., 2016). They also developed different bioinks to regulate the property of stem cell (Li Z. et al., 2018; Wang R. et al., 2019). Recently, Park’s group prepared a hybrid bioink composed of bioactive peptide-immobilized acrylated HA and tyramine-conjugated HAs (Lee J. et al., 2018). Results demonstrated that MSCs in these hybrid bioinks had high angiogenic and osteogenic activity. Bioprinting technology is also used to prepare a skin model *in vitro*. Kim et al. (2017) printed a 3D human skin model with a functional transwell system. Maturation of the skin model could be achieved with the support of printed PCL constructs (Kim et al., 2017). Kim’s group and Cho’s group also used skin-derived ECM as a bioink to fabricate a full thickness 3D skin, and the matured bioprinted skin tissue was significantly contracted during *in vitro* culture (Kim B. S. et al., 2018).

With this new method, a series of studies in which different materials (alginate, fibrin, gelatin, etc.) were performed to construct various tissues/organs (liver, adipose tissue, nerve, and so on) (Yang et al., 2013; Jung et al., 2016; Mishra, 2016; Igami et al., 2018; Liu F. et al., 2018). Other researchers also fabricated many kinds of cell/material complexes. Patterning of Sf-9 cell tissue was achieved using a mixture of cells and thermoreversible hydrogel (Iwami et al., 2010). Engineered human cartilage was prepared using an inkjet printer by printing poly(ethylene glycol) diacrylate (PEGDA) containing human chondrocytes (Cui et al., 2014). Gao G. et al. (2017) developed a 3D cartilage using bioprinting and simultaneous photopolymerization. This printed cartilage showed excellent production of glycosaminoglycan and collagen type II. In the ear reconstruction field, bioprinting is also widely used. For example, a tissue-engineered ear was prepared by printing PCL, cell-laden hydrogel, and sacrificial polyethylene glycol (PEG) layer (Lee et al., 2014a). The chondrocytes and adipocytes differentiated from ADSCs were encapsulated in hydrogel and distributed to the regions of cartilage or fat, respectively. The hydrogel could promote both chondrogenesis and adipogenesis of encapsulated ADSCs.

Functional vascular with perfusion performance could also be prepared using bioprinting cells and biological matrices (Lee et al., 2014b). This perfusable vascular can support the viability of tissue up to 5 mm in distance. Du et al. (2015) prepared BMSC-laden microfibers immobilized with BMP-2 by bioprinting. These prepared CBD-BMP-2-collagen microfibers induced osteogenic differentiation of BMSCs within 14 days more efficiently than the osteogenic medium. Jia et al. (2016) directly printed perfusable vascular constructs using a mixed bioink, composed of GelMA, sodium alginate, and 4-arm poly(ethylene glycol)-tetra-acrylate. These constructs could support the growth of encapsulated endothelial and stem cells. Finally, highly organized and perfusable vessels could be obtained. Jang et al. (2017) prepared a stem cell patch with pre-vascularized and functional multi-material structures by 3D printing using bioink consisting of cell-laden decellularized ECM (dECM), which promoted rapid vascularization after transplantation and enhanced the therapeutic efficacy for cardiac repair. This method was applied in many tissues including adipose, cartilage, and heart. The use of tissue-specific dECM in this process promised high cell viability and functionality (Pati et al., 2014).

Chang et al. (2010) fabricated a 3D liver micro-organ by the combining direct cell writing bioprinting process with micro-patterning techniques; this liver model could act as an *in vitro* model for drug metabolism. Scientists from Japan fabricated a liver model using the system “PLUTO.” The 3D-printed liver assisted the minor hepatectomy following liver partition (Igami et al., 2018). In hepatectomy for a small tumor that is invisible to intraoperative ultrasonography, the application of 3D-printed liver makes the procedure easy and feasible (Igami et al., 2014). Besides, 3D printing can also be used in fabricating other tissues. Scientists from South Korea and India printed a kidney, heart, and tooth, which presented the applications of 3D printing in regenerating heterogeneous organs and tissues to treat specific defects or injuries (Jung et al., 2016; Mishra, 2016).

#### Cell Sheet Technology

Cell sheet technology is a scaffold-free approach that has great potential in TERM (Kobayashi et al., 2019; Li M. et al., 2019). Compared with traditional cell therapies (cell suspension injection or cells/scaffold construct), cell sheet technology enables transplanted cells to stay completely in the target sites and fully maintain their viability. Since Okano’s group first devised a scaffold-free method of constructing cell sheets using thermoresponsive poly(*N-*isopropylacrylamide)-grafted surfaces, this technology has been applied to the regeneration of a variety of tissues (Takezawa et al., 1990; Sekine et al., 2013). In addition, it has also been used in cell micropatterning, cell co-culture, and drug discovery (Hannachi et al., 2009; Takahashi and Okano, 2019). Compared to other tissue engineering techniques, one of the major advantages of cell sheet is the absence of potential material degradation-associated cytotoxicity. Different methods have been developed to prepare cell sheets, including thermo-responsive systems, electro-responsive systems, photo-responsive systems, pH responsive systems, magnetic responsive systems, and so on (Inaba et al., 2009; Chen et al., 2012; Hong et al., 2013; Pan et al., 2013; Zhang W. et al., 2017; Koo et al., 2018; Li M. et al., 2019). Li’s group reported several new methods for cell sheet preparation (Pan et al., 2013; Guo B. et al., 2015). Compared with physical absorption and covalent immobilization approaches, the method through thermo-responsive specific binding RGD in the imprinted hydrogel not only promoted cell adhesion during cell culture but also facilitated cell detachment during cell sheet harvest (Pan et al., 2013). In the electricity-induced method and magnetic method, electricity-responsive thiol layers and pH-responsive layers have been designed to harvest cell sheets, respectively (Hong et al., 2013; Koo et al., 2018).

Cell sheet technology has been applied to treat damaged tissues including bone, articular cartilage, corneas, periodontal ligaments, blood vessels, and the myocardium (Takezawa et al., 1990; Sekine et al., 2013). Yang et al. (2007a) focused on using temperature-responsive surfaces to harvest cell sheets, and their applications in corneal dysfunction, tracheal resection, esophageal cancer, and cardiac failure. They have also created thick vascularized organ-like systems for the heart and liver based on cell sheet. To construct 3D tissues, some studies tried to prepare multilayered cell sheets. Most multilayered cell sheets were fabricated by layer-by-layer stacking individual cell sheet using a gelatin gel plunger or forceps (Haraguchi et al., 2012; Ren et al., 2014). Furthermore, 3D tissues can also be constructed via this protocol. At present, the cell sheet technique has already been applied to human clinical trials for regenerating heart, cornea, blood vessels, periodontal membrane, esophagus, cartilage, functional tendons, and middle ear (Egami et al., 2014; Yamamoto et al., 2017). For example, Yamamoto et al. (2017) developed a novel treatment method to regenerate the middle ear mucosa by tympanoplasty and autologous nasal mucosal epithelial cell sheet transplantation. All patients showed good regeneration result with no adverse complications and adverse effect on the patients’ hearing ability. Cell sheets composed of corneal epithelial cells or autologous oral mucosal cells could also repair severe corneal injury in patients (Nishida et al., 2004). In another study, transplantation of multilayered cell sheets composed of patients’ periodontal ligament derived MSCs was proved to promote regeneration of the periodontal and bone (Iwata et al., 2015).

#### Genome Editing

Recent advances in genomic technology and genome engineering make it possible to modify the genome in cells. A great progress of genome editing technologies, the CRISPR/Cas system (Komor et al., 2017; Kim, 2018), can directly modify the genome. Therefore, this technology can be used to systematically dissect the functional effect of genetic variants. Recently, there is much work done by Asian researchers (Duan et al., 2014; Liang et al., 2015; Kang et al., 2016; Xue et al., 2016). Gao et al. (2016) determined the mechanism of CRISPR-Cpf1 system, by analyzing the structure of *Acidaminococcus* sp. Cpf1 in complex with crRNA and target DNA. Recently, Zhou et al. (2018) improved the Cas9 system to be much precise and could simultaneous activate multiple genes and long noncoding RNAs in the nervous system.

The safety of technology is the primary issue to be considered (Pineda et al., 2019). One of the safety concerns for CRISPR technology is unforeseen genetic, which may bring unpredictable physiological changes or even death to patients. The off-target genomic and cellular events are also scientists’ concerns. In addition, mutations in vital genes could cause cancer or the damage of organs. Hence, the safety evaluation for CRISPR technology needs to advance in parallel with CRISPR technologies. To improve the safety and efficiency of CRISPR/Cas9 gene-editing elements, Leong’s groupreported an efficient CRISPR/Cas9 delivery system comprising PEGylated nanoparticles based on poly (γ-4-((2-(piperidin-1-yl)ethyl)aminomethyl)benzyl-L-glutamate) for delivering Cas9 expression plasmid and sgRNA (Wang H. X. et al., 2018). This CRISPR/Cas9 delivery system could reach 47.3% gene editing efficiency in single or multiplex gene editing *in vitro*, achieve 35% gene deletion in HeLa tumor tissue, suppress the tumor growth by >71%, and prolong the animal survival rate to 60% within 60 days. Peng’s group analyzed the function and structure of two archaeal anti-CRISPR proteins (Acrs) from the lytic rudiviruses, SIRV2 and SIRV3 (He et al., 2018). Tan’s group developed a Cas9 variant whose activity can be switched on or off in cells by 4-hydroxytamoxifen; this Cas9 variant showed high gene editing efficiency and could reduce 437 off-target cleavage (Liu et al., 2016). Dong et al. (2017) revealed the mechanism of SpyCas9 inhibition by AcrIIA4, which make it possible to develop “off-switch” SpyCas9 systems to avoid unwanted genome edits within cells and tissues.

#### Microfluidics

In the past two decades, various cell microencapsulation technologies have been developed (Alkayyali et al., 2019). Cells encapsulated in microbeads or microfibers could be prepared by changing microfluidic devices or materials. The flexibility of microfluidic technology allows the preparation of multi-structured fibers such as hollow, multilayer, grooves, etc. Yu et al. (2017) prepared alginate helical microfiber with complex structures such as Janus, triplex, core–shell, and even double-helix based on rapid ion cross-linking through microfluidic devices. In recent years, the application of microfluidics in cell-laden droplet production and single-cell encapsulation has been reviewed (Zhu and Yang, 2017; Alam et al., 2018; Alkayyali et al., 2019; Shen et al., 2019). Zhao’s group prepared uniform microfibers by simple injection capillary microfluidics, core–shell or spindle-knot structured microfibers by hierarchical injection capillary microfluidics, and microfibers with multiple components by multi-barrel injection capillary microfluidics (Wang J. et al., 2017; Yu et al., 2017; Yu Y. et al., 2018). A cell microcarrier with controllable macropores and heterogeneous microstructures could also be fabricated by a capillary array microfluidic technology (Wang J. et al., 2017). These microcarriers with spatially heterogeneous cell encapsulations may be used for mimicking physiological structures of nature tissues or organs. Lee’s group encapsulated *Escherichia coli* (*E. coli*) in PEGDA microdroplets using a microfluidic device (Lee K. G. et al., 2010). This encapsulated *E. coli* may be used in biotransformation, bioremediation, biosensing, and artificial cells. Jia et al. (2019b) prepared biomimetic cell-laden helical hydrogel microfibers as blood-vessel-on-chip using alginate based on microfluidics methodology. This hollow microfiber had decent perfusability and could generate swirling blood flow to provide a platform for mimicking nature swirling blood flow. Except cell encapsulation, the applications of microfluidics in single molecule/cell culture and analysis (Zhu and Yang, 2017), cell sorting (Shen et al., 2019), continuous cell separation (Yan et al., 2017), and orthopedics (Wang L. et al., 2019) were also reported in some reviews.

#### Organ-on-a-Chip

Bridging the gap between findings in 2D cell culture *in vitro* and 3D tissue culture condition *in vivo* has been a challenge. 3D printing and microfluidic devices also offer great development of an *in vitro* model in TERM, organ-on-a-chip (Kim et al., 2013; Bang et al., 2017; Lee and Jun, 2019; Mobini et al., 2019; Ranjith Kumar et al., 2019; Sun W. et al., 2019). Jeon’s group reported several work in this field (Kim et al., 2013; Bang et al., 2017). They constructed a low-permeability blood–brain barrier platform by microfluidics (Bang et al., 2017). Using this platform, co-culturing human umbilical vein endothelial cells and neural cells with independent culture medium could be easily realized. In another work, they modeled natural cellular programs through a microfluidic-based platform, and using this platform, they could spatially control co-culture of endothelial cells with stromal fibroblasts, pericytes, or cancer cells (Kim et al., 2013). The lab-on-a-chip devices were also used to construct *ex vivo* models for neural tissue engineering (Mobini et al., 2019). The group of He fabricated 3D hydrogel-based vascular structures with macrochannels (Gao Q. et al., 2017). These 3D hydrogel-based vascular structures could be further integrated into organ-on-chip devices to better simulate the microenvironment of blood vessels. In addition, the organ-on-a-chip also has great potential in preclinical testing of drugs (Ranjith Kumar et al., 2019).

### Mechano-Regulation in TERM

The organism in a complex mechanical system consists of external and internal forces. Cells, tissues, and even organs are stimulated by mechanical stimuli of different intensity, frequency, and direction. Mechanical stimuli regulate cell function by affecting gene expression and protein synthesis in cells and then regulate cell differentiation, growth, and development of organisms. Therefore, mechanical stimuli also play an important role in TERM. This unique form of mechanical force is regulated in real time by a variety of factors, such as exogenous force, motor proteins, osmotic pressure, mechanosensitive ion channels, intracellular mechanosensors, and actin assembly. Traditional approaches in cellular mechanobiology include loading tissues or organ culture *in vivo*, or loading cells in monolayer and scaffolds *in vitro* at various modes, magnitudes, and frequencies of mechanical stimuli. In the past decades, the field of mechanoregulation of tissue engineering and regeneration medicine has witnessed tremendous progress in Asia.

#### Bone

Mechanical stimulus is one of the most important physical stimuli for bone, which is known to regulate the osteogenic differentiation of MSCs and other osteogenic precursor cells. However, the various modes of stimuli showed different effects on osteogenesis. For example, cyclic mechanical tension inhibited Runx2 expression in MSCs through the RhoA-ERK1/2 pathway (Shi et al., 2011). In a study, 1 Hz cyclic mechanical stretch for 30 min (twice a day) could enhance osteogenic differentiation of BMSCs and restrain osteoclastogenesis of RAW264.7 cells *in vitro* (Guo Y. et al., 2015). In another study, 2 h of cyclic stretch of 2.5% elongation at 1 Hz on 3 days significantly decreased osteogenesis of adult MSCs by the increased reactive oxygen species (Tan et al., 2015). The mechanical stretch could also affect antioxidant responses and osteogenic differentiation in human MSCs through activation of the adenosine 5′-monophosphate-activated protein kinase–Silent mating type information regulation 2 homolog-1 (AMPK-SIRT1) signaling pathway (Chen et al., 2018b). Additionally, exercise can strengthen bones (Niinimaki, 2012). Exercise was proved to inhibit bone loss in ovariectomized rats (Bu et al., 2012). The expression of Runx2 was reported to gradually increase under strain stimulation (Li C. J. et al., 2015; Li R. et al., 2015). The same result was also found in another study in rats (Notomi et al., 2014). In conclusion, suitable exercise may promote osteogenic differentiation of MSCs by upregulating the expression of key molecules in Wnt signaling pathway.

#### Tendon and Ligaments

Mechanical stimuli play an important role in regenerating or repairing tendons and ligaments. Mechanical stimuli can enhance cell proliferation and tenogenic differentiation of tendon-derived stem cells (TDSCs). In a study, the TDSCs-poly(LLA-CL)/Col scaffolds were stretched at 4% elongation, 0.5 Hz, and 2 h per day for 14 days, the mechanical stimuli could promote formation of tendon tissues after the implantation of TDSCs-poly(LLA-CL)/Col scaffolds in nude mice (Xu et al., 2014). This result showed that dynamic mechanical stimulus was helpful for the maturation of tissue-engineered tendon *in vivo*. In another study, the mechanical stretch (10% elongation for 48 h, 10 cycles/min, each cycle containing 2 s of stretch, and 2 s of relaxation) accelerated the healing of tendon–bone by promoting proliferation and matrix production of MSCs and tendon cells (Song et al., 2017). However, there was also a report that the cyclic stretch could induce apoptosis of human periodontal ligament cells via a caspase-dependent pathway (Chen J. et al., 2016; Wu Y. et al., 2016; Zhao D. et al., 2017). In another study, the cyclic tensile promoted BMP-9 synthesis and *in vitro* mineralization in human periodontal ligament cells (Tantilertanant et al., 2019). The study of mechanical stimuli on tendon/ligament repair has been extensively studied with variable degrees of efficacy. In the future, synergistic therapies for tendon/ligament repair and regeneration may be created by combining mechanical loading with biochemical cues.

#### Cartilage

Due to its avascular nature and limited proliferative potential of mature chondrocytes, cartilage only has limited intrinsic regeneration ability. Mechanical stimuli have been shown to affect the gradient of nutrient and ion, pH, cell deformation, bioactivity, and synthesis of ECM components or growth factor (Bleuel et al., 2015). Different levels of shear stress could affect the expression of inflammatory stimuli-induced genes in chondrocytes through macrophage-induced c-Jun N-terminal kinase and Akt phosphorylation, nuclear factor κ-B activation, and urokinase plasminogen activator expression (Yeh et al., 2013). The compressive stress may promote chondrogenic differentiation by increasing the activity of TGF-β1 and regulating the downstream targets of TGF-β signaling. It was reported that Smad pathways would be involved in the conversion of mechanical stimuli into biological responses (Li J. et al., 2012). While the physiological level of mechanical stimuli helps the maintenance of healthy tissues, excess mechanical stimuli result in damage of tissues. Koike et al. (2015) demonstrated that excess mechanical stimuli promoted mitochondrial superoxide generation and caused imbalance of superoxide dismutase 2 in chondrocytes, which would result in cartilage degeneration. Recently, Iran researchers constructed a new microdevice used to study chondrogenesis under unidirectional compressive stimulus of cells in a 3D cell culture condition (Kowsari-Esfahan et al., 2018). The results showed that the dynamic mechanical compression enhanced cell viability and upregulated the expression of Sox-9, collagen II, and aggrecan in the absence of exogenous growth factors. Moreover, 10% strain was considered as optimal mechanical stimulus for chondrogenic differentiation of ADSCs.

#### Cardiac Tissue

Regenerating the beating heart is still a formidable bioengineering challenge. The pumping action of the heart requires mechanical forces to compress a blood-filled chamber with a defined in- and outlet. It is widely accepted that mechanical loads are important in the development and morphogenesis of cardiac tissue. In a study, it was demonstrated that fluid shear stress would affect cardiomyogenic differentiation of rat BMSCs (Huang Y. et al., 2010). Zhang W. et al. (2017) prepared cardiac muscle strips by encapsulating cardiomyocytes derived from human embryonic stem cells in collagen-based biomaterials. The results demonstrated that the mechanical stretch could promote the maturation of human embryonic stem cell-derived cardiomyocytes. Cyclic biaxial tensile strain promoted the differentiation of BMSCs into cardiomyocyte-like cells by miRNA-27a (Cao et al., 2018).

#### Intervertebral Disc

Intervertebral disc (IVD) not only absorbs the mechanical load but also maintains multi-axial flexibility of the spine. The mechanical disorder of IVD will ultimately cause tissue dehydration, fibrosus, nerve, and vessel ingrowth, and disc degeneration (Chu et al., 2018; Li and Chen, 2019). For instance, the increasing frequency and amplitude of dynamic compress would contribute to higher cell density, because dynamic compression facilitated the diffusion of nutrients around IVD cells (Zhu et al., 2012). But another study showed that static compression significantly decreased the density of nucleus pulposus (NP) cell, but increased TUNEL-positive cells and apoptosis index (Kuo et al., 2014). Moreover, this effect is in a dose-dependent manner. It was thought that the intrinsic (mitochondrial) apoptotic pathway played an important role in compression-induced NP cells apoptosis. Further research showed that the dynamic compressive load reduced the density and viability of NP cells. The localization and mRNA expression of integrin α5β1 were increased in both NP and annulus fibrosus (AF) under dynamic mechanical stimuli. Dynamic compression load (1.3 MPa, 1.0 Hz) also showed a catabolic effect, and the expression of matrix metalloproteinase-3 and metalloproteinase-13 in NP and AF cells would be regulated by dynamic mechanical stimuli (Kurakawa et al., 2015).

#### Blood Vessels

The mechanical stimuli also play an important role in blood vessels. The suitable cyclic stretch also induced vascular remodeling by promoting VSMC proliferation. In a study, the physiological cyclic stretch promoted the contractile differentiation of VSMCs via the SIRT1/FOXO pathways and thus maintained vascular homeostasis (Huang et al., 2015). Mechanical stretch-induced endoplasmic reticulum stress could also promote the apoptosis, inflammation, and degeneration of VSMCs (Jia et al., 2015).

## Applications of Term in Different Tissues

### Cartilage

The repair and regeneration of cartilage, such as ear and joint, are still associated with various limitations and degrees of success (Bauer, 2009; Lee T. S. et al., 2010; Tang et al., 2017). TERM provides us a new way to construct engineered cartilage substitutes, which could match both the function and appearance of native ears (Peer, 1954; Watson, 2009). By combining suitable cell source with scaffolds, engineered cartilage constructs could replace injured tissues. Since 1997, Cao and colleagues have used engineered cartilage to treat cartilage diseases. They found that both component and structure of the scaffolds would affect cartilage regeneration. Cao and Zhou’s groups successfully regenerated ear-shaped cartilage combining electrospun gelatin/PCL membranes and cells (Zheng R. et al., 2014). Further, they successfully regenerated subcutaneous cartilage using BMSCs directed by chondrocyte sheet (Li et al., 2017). Liu and Zhou’s groups created a human-ear-shaped cartilage tissue by seeding the microtia chondrocytes and BMSCs into the ear-shaped biodegradable scaffold and subcutaneously implanting them into nude mouse, which provides a promising strategy to construct stable ectopic cartilage (Zhang L. et al., 2014). He and Fu’s groups fabricated the ear tissue using a method named scanning printing polishing casting. They designed a mold according to the 3D scanner of ear and generated ear tissue by casting medical grade silicone to the mold. This strategy provided a method that has a lower cost than the current soft prostheses fabrication methods (He et al., 2014). Cho’s group generated an artificial ear containing auricular cartilage and fat tissue using 3D printing technology. In this process, ADSCs were differentiated to chondrocytes and adipocytes, and then encapsulated in hydrogel, respectively. The ear tissue was printed with PCL and cell-laden hydrogel to ear-shaped structures (Lee et al., 2014a). Hwang et al. (2014) spray-coated a mixture of chondrocytes and fibrin hydrogel on a human ear-shaped implant and implanted it into the dorsal subcutaneous space of athymic mice. The formation of neocartilage covered the implants after 12 weeks.

Successful regeneration of critical-sized cartilage defects in weight-bearing region remains a major challenge in clinic. In the past decades, biomaterials aiming to repair cartilage have been rapidly developed (Zhang Y. B. et al., 2019). Zhang’s group developed injectable carboxymethylated pullulan/chondroitin sulfate hydrogel and collagen I/II composite hydrogel for cartilage tissue engineering (Chen F. et al., 2016; Yuan L. et al., 2016). Yu’s group created chitosan microspheres to carry cells, and this system could be used for cartilage tissue engineering (Zhou et al., 2016). The chitosan microspheres showed an ECM-mimicking nanofibrous structure and tunable size could be constructed into 3D-shaped cartilage-like composite *in vitro*. Collagen is a widely used material in cartilage tissue engineering. To improve the unmatched stiffness and rapid degradation rate of collagen, Lu Z. et al. (2019) conjugated biocompatible carbon dot nanoparticles onto collagen to prepare an injectable hydrogel. This injectable composite hydrogel combined with PDT enhanced chondrogenesis. To encapsulate chondrocytes in scaffolds to promote cartilage regeneration, Wang C. Y. et al. (2019) prepared an injectable cholesterol-enhanced stereocomplex polylactide thermogel; the modification of cholesterol on the 4-arm PEG-PLA could not only promote the preservation of morphology and biomechanical property but also promote cartilaginous gene expressions. Ding’s group found that nanoscale spatial arrangement of RGD peptides could affect the dedifferentiation of chondrocytes (Li S. Y. et al., 2015). To realize the controlled chondrogenic differentiation, Wu’s group successfully regulated the anti-apoptotic gene and chondrogenic regulator using a double duration inducible gene expression system (Ma Y. et al., 2016). Ouyang’s group successfully used chondrocyte-derived progenitor cells to repair large knee cartilage defects of patients (Jiang et al., 2016). In another work by Ouyang’s group and Wu’s group, they used a silicate-based bioceramic scaffold with the ability to regulate cartilage and bone dual-lineage regeneration and repaired the osteochondral defect (Bunpetch et al., 2019). Recently, Fan and Wang’s groups constructed an acellular matrix microsphere as a scaffold for engineering cartilage (Liu J. et al., 2019). Park’s group and Min’s group used decellularized porcine cartilage-derived ECM to fabricate scaffolds (Oh et al., 2018). This ECM-derived scaffold could provide suitable environments for chondrocyte growth. In another work, they repaired human partial thickness cartilage defects using a cartilage ECM membrane that can deliver chondrocytes (Park et al., 2016). Ha’s group used human umbilical cord blood-derived MSCs to repair cartilage of rats and obtain favorable results (Park Y. B. et al., 2019). In a clinical trial, they successfully regenerated articular cartilage in the defects of osteoarthritis patients, using a composite of allogeneic umbilical cord blood-derived MSCs and hyaluronate hydrogel (Park Y. B. et al., 2017).

### Skin

The development of artificial skin has attracted a lot of Asian researchers. Various skin wounds will be caused by burn, freezing, surgical procedures, radiation, and chronic skin ulcers. The skin substitutes prepared by tissue engineering could be used to promote acute and chronic skin wound healing (Tang et al., 2017). Several Asian research groups have made great efforts in skin tissue engineering. Jin’s group developed several multilayered skin substitutes (Zhu et al., 2008; Wang et al., 2009; Huang S. et al., 2010). For instance, they prepared a tricopolymer scaffold consisting of collagen, chondroitin sulfate, and HA to mimic ECM of the skin (Wang et al., 2009). Further, they also attempted different cell sources for skin repair, including BMSCs, melanocytes, keratinocytes, dermal fibroblasts, human amniotic epithelial cells, human amniotic mesenchymal cells, and adipose tissue-derived stem cells (Liu et al., 2007, 2014; Li H. et al., 2012; Lu et al., 2012; An et al., 2015). Based on these works, Jin’s group first developed tissue-engineered artificial skin in China. Fu’s group has constructed different skin substitutes by tissue engineering. A collagen-based and cell-seed skin construct was prepared by culturing sweat gland cells on gelatin microspheres and then combining them with collagen (Huang S. et al., 2010). This artificial skin construct promoted skin repair and wound healing. Further, they designed a skin scaffold with modifying spatial inductive cues by bioprinting, which could promote differentiation of epidermal lineages into sweat glands (Huang et al., 2016). In addition, they also tried various cells for skin tissue engineering (Li H.-H. et al., 2008, 2009). Ruszymah’s group did some studies on skin tissue engineering (Chowdhury et al., 2015; Law et al., 2016, 2017; Fauzi et al., 2019). They found that skin cells, keratinocytes, and fibroblasts showed different cellular activities within collagen and fibrin constructs (Law et al., 2016). Co-culturing keratinocytes with fibroblasts in the collagen scaffold could reduce the proliferation of fibroblast and collagen contraction. In another study, they demonstrated that dermal fibroblast conditioned medium promoted skin wound healing (Chowdhury et al., 2015). In addition, they found that plasma-derived fibrin could promote wound healing (Law et al., 2017). Recently, they reported rapid repair of full-thickness skin injury using ovine tendon collagen I scaffold combining with skin cells (Fauzi et al., 2019).

Until now, different types of scaffolds, including the nanofibers (Xu et al., 2019; Yao et al., 2019; Zhang K. X. et al., 2019), hydrogels (Hsu et al., 2019; Muthuramalingam et al., 2019; Patel et al., 2019), porous scaffolds (Wang et al., 2009), particles (Shi M. et al., 2019), decellularized bioscaffolds (Cui et al., 2019), and bioadhesives (Deng et al., 2019; Park E. et al., 2019) have all been applied in skin repair. The antibacterial conductive hydrogels fabricated by supramolecular assembly of PDA-modified silver nanoparticles, polyaniline, and polyvinyl alcohol could be used for epidermal sensors and diabetic foot wound dressings (Zhao Y. et al., 2019). A β-glucan-based hydrogel significantly not only accelerated wound healing but also promoted the regeneration of skin appendages (Muthuramalingam et al., 2019). To eliminate the undesired immune responses in practical use of chitosan–catechol in the clinic, a catechol-conjugated glycol chitosan adhesive hydrogel was proposed as an alternative and exhibited negligible immune responses (Park E. et al., 2019). Another bioinspired adhesive derived from skin secretion of *Andrias davidianus* showed stronger tissue adhesion strength than fibrin glue, which may be used for wound healing. Moreover, the elasticity and biocompatibility of this adhesive were better than those of cyanoacrylate glue (Deng et al., 2019). The addition of bioactive substances, such as the grape-seed extracts containing rich flavonoids with oligomeric proanthocyanidins, could promote wound healing (Ma et al., 2019). The multifunctional Zn-doped hollow mesoporous silica/PCL electrospun membranes with good antibacterial activity were shown to enhance hair follicle regeneration and wound healing (Zhang Y. et al., 2019). The electrospun poly(gamma-glutamic acid) fibrous scaffolds decorated with ginsenoside Rg3 could accelerate fibroblasts to sprout and grow, promote scar-free wound healing *in vivo*, and prevent hypertrophic scars (Xu et al., 2019). In a recent study, researchers combined 3D printing of poly (L-lactide-co-caprolactone) scaffold with bioactive peptide hydrogel and showed a good treatment modality in treating skin defects (Im et al., 2018). In a recent study, Koo et al. (2019) transplanted a three-layered cell sheet stacked by ROS-induced method which took only a short time. The results showed that the stacked cell sheet promoted angiogenesis and skin regeneration.

### Bone

The aim of bone tissue engineering is to develop ideal bone substitutes (Shrivats et al., 2014). Generally, these substitutes contain cells and should support cellular behaviors and guide bone regeneration. Yanaga et al. (2006) made an attempt to use a composite of autologous human auricular chondrocytes with autologous serum for craniofacial augmentation in a pilot clinical study. Otsu’s group revealed that neural crest-like cells from iPS cells could differentiate into osteoblasts and contribute to craniofacial bone regeneration without tumor formation *in vivo* (Kikuchi et al., 2018). This method could resolve the limitation of insufficient source of reconstructive material. Cui’s group developed various scaffolds to mimic the natural bone ECM niche. They developed a nano-HAp/collagen/PLA composite (Liao et al., 2004) or constructed a porous bone scaffold (Li X. et al., 2006). They also added the mineralized collagen into poly(methyl methacrylate) (PMMA) bone cement (Li T. et al., 2015). Now, they have developed a variety of artificial bone repair materials, which acquired the product registration certificate from the Chinese government. Cho’s group and Lee’s group prepared a synthetic scaffold with patient-customized structure and mechanical properties for bone repair (Lee et al., 2019). Aiming to repair bone defects with irregular shapes, Wang L. H. et al. (2019) recently developed 3D super-elastic scaffolds based on electrospun SiO_2_ nanofibers with self-fitting and tailorable gradient capability. Lin’s group used a HAp–calcium sulfate–HA composite carrying collagenase to repair alveolar bone (Subramaniam et al., 2016). Dewi et al. (2015, 2017) added calcium carbonate into plaster of Paris to improve the mechanical and degradable property of the bone substitutes. In addition, Wu’s group also prepared lithium-containing bioactive glass ceramic, which promoted angiogenesis for bone tissue engineering (Liu L. et al., 2019), and an injectable sodium alginate/bioglass composite hydrogel containing BMSCs for subchondral bone regeneration (Zhu et al., 2019). Kim and Yoo’s groups first reported that immobilizing BMP-2 into hydrogel promoted osteogenic differentiation of human periodontal ligament stem cells (Park S. H. et al., 2017). Liu’s group promoted rapid bone regeneration by spatiotemporal delivery of BMP-2 or interleukin-8 from scaffolds (Chai et al., 2017; Lin D. et al., 2019). Qin’s group designed a targeted delivery system that could release osteogenic phytomolecule icaritin to prevent osteoporosis in a bone-targeting manner (Huang et al., 2018). Wang’s group used allogeneic mesenchymal stromal cell with low immunogenicity to construct bone substitutes and to repair bone defects in pigs (Chen Q. et al., 2010; Ren et al., 2012). In addition, they also found the function of modulating immunity and inducing immune tolerance of allogeneic mesenchymal stromal cells (Peng et al., 2012). Likewise, growth factors such as BMP-2 and TGF-β1 and vascular endothelial growth factor (VEGF) are also introduced to scaffolds to enhance bone repair (Qu et al., 2015; Yan et al., 2016). Yang’s group developed PLGA/PCL scaffold modified by silver impregnation, collagen coating, and electrospinning; this scaffold showed osteogenic and antimicrobial properties for orofacial tissue regeneration (Qian et al., 2019). Chua et al.’s (2004) group prepared polyvinyl alcohol/HAp biocomposite by selective laser sintering; this material showed good potential for craniofacial repair. Due to their cortical bone-like mechanical properties, biometals may become potent options for bone scaffolds. Among them, Mg has attracted a lot of attention of researchers (Zhang et al., 2009; Lai et al., 2019). Dai’s group and Chang’s group have prepared calcium Mg silicate bioceramic scaffolds and found that the scaffolds enhanced angiogenesis in bone regeneration (Huang Y. et al., 2009; Zhai et al., 2012). Yang’s group evaluated the *in vivo* degradation of Mg alloy implant and showed more new bone tissues around the implant after 10 and 26 weeks post-implantation, while only causing little change to blood composition (Zhang et al., 2009). They also developed various metal implants for bone tissue engineering (Li Y. et al., 2016; Zhang X. Z. et al., 2017). Qin’s group investigated the osteogenic capacity of Mg and found that PLGA/TCP porous scaffold incorporated Mg by 3D printing and had better repair result in challenging bone defect (a 12-mm segment of ulna in rabbit) (Lai et al., 2019).

### Cornea

The development of corneal repair and regeneration is a pressing issue in TERM. Zhao X. et al. (2019) introduced gold nanoparticle-loaded microRNA-133b into collagen membrane. These collagen membranes could rapidly repair corneas and effectively inhibit scar formation. Hashimoto et al. (2019) realized the re-epithelialization and remodeling of rabbit decellularized corneal matrix, suggesting that this material may be a useful graft for corneal tissue regeneration. Developing a synthetic scaffold with similar properties to native cornea remains a challenge. In a study, the researchers developed elastomeric and biodegradable poly(glycerol sebacate)-PCL nanofibrous scaffolds and they show similar optical and mechanical properties with cornea (Salehi et al., 2017). Choi et al. (2018) also used biofunctionalized lysophosphatidic acid/silk fibroin film for corneal regeneration; this film showed higher specific gene and protein expression of cornea endothelial cells. Lee H. J. et al. (2018) used an *in situ* forming corneal stromal substitute based on collagen type I, after encapsulating keratocytes; this substitute exhibited surface epithelialization with multilayered morphology when implanted to rabbit corneas in an organ culture model after keratectomy. The researcher also tried to use short collagen-like peptides (CLPs) to replace collagen in corneal tissue engineering. They found that CLP-modified implants promoted stable regeneration of corneal and nerve tissues in a mini-pig model (Jangamreddy et al., 2018). Cells were used to promote corneal regeneration. For example, Yamashita et al. (2018) found that MSCs derived from human umbilical cord transplanted into a rabbit could maintain the thickness and transparency of cornea. Cell sheets are also used to repair cornea. Okano’s group successfully regenerated cornea in rabbit by transplanting corneal epithelial cell sheets prepared with automated cell culture system (Kobayashi et al., 2013). Later, they reported a novel cell culture device for preparation of corneal epithelial cell sheets (Nakajima et al., 2015). Fujita’s group transplanted an autologous corneal epithelial cell sheet in corneal injury of canine and demonstrated that the cell sheet may restore corneal transparency and prevent irreversible opacity caused by severe injury (Nam et al., 2018).

Corneal stroma provides the principal functions of the cornea. Tissue-engineered corneal stroma will be a promising strategy to overcome donor shortage in cornea replacement. Jin’s group attempted to implant a corneal stroma by seeding rabbit stromal keratocytes onto decellularized porcine cornea (DPC) into a model of corneal ulcer (Zhang et al., 2007). Recently, they used an artificial cornea composed of amniotic epithelial cells and acellular porcine cornea to treat corneal alkali burn (Luo et al., 2013). Cao’s group demonstrated that a polyglycolic acid (PGA) scaffold loading corneal stromal cells successfully repaired cornea (Hu et al., 2005). Moreover, they also reported a composite corneal scaffold containing acellular corneal stroma sheets and keratocytes (Ma et al., 2015). Wang’s group fabricated engineered lamellar cornea by seeding genetically modified embryonic stem cells and corneal epithelial cells on decellular porcine corneal stroma and amniotic membrane (Zhou Q. et al., 2014). The results showed that this substitute promoted wound healing and showed epithelial barrier functions. Recently, Xie and Shi’s groups developed a new method to prepare DPC grafts, which could efficiently remove the major xenoantigen but preserve corneal original structural and transparency (Shi W. Y. et al., 2019). These DPC grafts showed similar epithelial regeneration rate, transparency restoration, and visual acuity improvement when using human donor cornea grafts. Cells were also introduced into developing tissue-engineered corneal stroma. A cell-laden and orthogonal-multilayer tissue-engineered corneal stroma facilitated the construction of physiological feature tissue-engineered corneal stroma and helped to reverse fibrosis pathologies in general (Cui et al., 2018).

### Nerve

The regeneration of traumatic and degenerative central or peripheral nervous system (CNS or PNS) injuries is still a big challenge. The CNS of adult mammalian is difficult to regenerate by itself, while the PNS only has limited axonal regeneration ability (Gu et al., 2011). Tissue-engineered neural scaffold may help to repair SCI or brain injury. Li’s group developed a neurotrophin-3 (NT-3)-containing chitosan-based scaffold, which could promote axon regeneration and functional recovery of SCI or brain injury (Li et al., 2009; Mo et al., 2010). In another work, NT-3-containing chitosan-based scaffold could elicit activation of endogenous NSCs, enhance neurogenesis and vascularization, and reduce inflammatory responses, thus improving the recovery of sensory and motor behavior (Duan et al., 2015; Yang Z. et al., 2015). Recently, they have obtained good repair results using the NT-3-containing chitosan scaffold in monkeys after SCI (Rao et al., 2018). Dai’s group developed collagen-based neural scaffold to promote axonal and spinal cord regeneration in canine and rodent models (Duan et al., 2015; Li X. et al., 2015). They also combined scaffold with BMSCs to construct a collagen/BMSCs construct, which showed good repair result for severe uterine injury in rats (Ding et al., 2014). In a recent study, Dai’s group implanted linear-ordered collagen scaffold (LOCS) and LOCS combined with collagen binding NT-3 (CBD-NT-3) into an acute thoracic complete transaction model in rhesus monkeys, which is closer to humans (Han S. et al., 2019). Ten months after surgery, both the LOCS and LOCS + CBD-NT-3 groups facilitated the ingrowth of axonal fibers at the lesion site. Moreover, the LOCS + CBD-NT-3 displayed a significant locomotor and electrophysiological recovery effect compared to other groups. Zeng’s group also loaded NT-3 in gelatin sponge scaffold, which facilitated the regeneration of spinal cord in dogs or rats (Li G. et al., 2016). In other studies, they attempted to combine BMSCs with scaffolds (Zeng et al., 2011, 2016). For instance, they found that the BMSC-seeded gelatin scaffold could attenuate inflammation, promote vascularization, and reduce the formation of cavity in the injured spinal cord of rat (Zeng et al., 2011). In another study, BMSCs and Schwann cells combined with gelatin scaffold could induce neurite elongation and promote the regeneration of nerve fiber in SCI (Zeng et al., 2016). Cui’s group and Xu’s group have made an attempt to use HA-based scaffolds for repair or regeneration of CNS. They found that the HA-poly-L-lysine/nogo-66 receptor antibody composite scaffold could enhance axonal regrowth (Wei et al., 2010). Further, they demonstrated that the modification of laminin or Ile–Lys–Val–Ala–Val peptide in HA hydrogel could promote the regeneration of nerve (Hou et al., 2005; Wei et al., 2007). In addition, the scaffolds with different structures were also prepared for SCI repair. In a work, multi-channel PLLA conduits with nano-fibrous channel walls promoted directional growth of nerve fiber within the channels compared to multi-channel PLLA conduits with ladder-like porous channel wall (Sun X. et al., 2019). Other composite hydrogels consisting of polyacrylamide, graphene oxide, gelatin, and sodium alginate were also developed for repairing SCI (Chen S. et al., 2019). The photoimmobilization of protein on the inner surface of poly(2-hydroxyethyl methacrylate) hydrogel could further improve the adhesion and survival of NSCs in the conduit, and exhibited the sustainable release of basic FGF (bFGF) (Cai et al., 2019). This hydrogel created favorable biologic niches to promote SCI repair. A variety of fibrous scaffolds were also used in neural tissue engineering (Li Y. et al., 2019; Ye et al., 2019a). Chew’s group used poly(ε-caprolactone-co-ethyl ethylene phosphate) electrospun nanofibers–collagen hybrid scaffold to repair SCI (Nguyen et al., 2017). They reported that the aligned nanofibers could enhance axonal regeneration in SCI (Chew et al., 2015). Further, they used a hybrid scaffold composed of aligned nanofibers and hydrogel, which could deliver drug or gene to direct axon regeneration in SCI (Nguyen et al., 2017). In addition, Kwon’s group introduced PLA nanorods to improve the mechanical property of agarose hydrogel-based integrated neuro-electrodes, making it a useful material in neural tissue engineering (Heo et al., 2017). Okano’s group has done some clinical trials in human for treating SCI using iPSC-derived neural precursor cells (iPSC-NPCs) (Isoda et al., 2016; Nagoshi and Okano, 2018; Tsuji et al., 2019). They have successfully generated NPCs from iPSCs and proved their function and safety (Isoda et al., 2016; Nagoshi and Okano, 2018). At present, 2 × 10^6^ iPSC-NPCs was transplanted into the SCI patients at 14–28 days after injury and followed-up for 1 year.

There are also many efforts to promote the repair of peripheral nerve and clinical translation of tissue-engineered products (Gu, 2015). Gus’s group has long contributed to use biomaterials for peripheral nerve regeneration (Gu X. et al., 2014). For example, they reported work using silk fibroin and chitosan for peripheral neural tissue engineering (Wu et al., 2005; Yang et al., 2007b). They further engineered chitosan- and silk fibroin-based neural scaffolds by seeding BMSCs and implanted them in rats (Yang et al., 2011), dogs (Xue et al., 2011), and rhesus monkeys (Hu et al., 2013). Successful nerve repair and functional recovery indicated the therapeutic effect of combining scaffolds and BMSCs. In addition, they identified a bioactive molecule (achyranthes bidentata polypeptides) from a traditional Chinese medicine (Cheng et al., 2014). This molecule could promote repair of peripheral nerve like as growth factors. In recent studies, Gu Y. et al.’s (2014) group proposed that it was important to improve and proved to be regenerative within a neural scaffold. For example, the degradation products from chitosan scaffold may facilitate the regeneration of peripheral nerve by improving macrophage-constructed microenvironments (Zhao Y. H. et al., 2017). Gu’s group also promoted the clinical translation of neural scaffolds in China (Hvistendahl, 2012). In a report, they repaired two patients by chitosan-based scaffolds, and the 3-year follow-up displayed good functional recovery of injured nerves (Fan et al., 2008; Gu et al., 2012). Many other nerve grafts were also developed (Tao et al., 2019; Wang J. et al., 2019). Mo’s group fabricated a composite nanomaterial by coating bioactive graphene oxide on nanofibrous scaffold for successfully repairing a sciatic nerve defect (10 mm) in rats (Wang J. et al., 2019). A drug-loaded PEG–PCL nanoparticle in GelMA hydrogels prepared by 3D printing showed effective nerve repair (Tao et al., 2019). Other acellular nerve grafts were also prepared. A joint research by Lu and Wang’s groups reported novel 3D helix-flexible nerve guide conduits used for nerve repair (Quan et al., 2019). This style of helix-flexible nerve guide conduits could be used to repair peripheral nerve in a cross-joint region due to the lower tension during operation and easy rehabilitation after operation. Lu and Peng’s groups developed acellular nerve grafts transfected with hepatocyte growth factor to repair rat sciatic nerves (Li Z. et al., 2008). Recently, they combined acellular cauda equina allograft with chitin to repair long-distance sciatic nerve defect in rats (Zhao Y. H. et al., 2017). They also combined different cells, including BMSCs (Zhao et al., 2011), BMSC-containing fibrin glue (Zhao Z. et al., 2014), and Schwann cell-like cells derived from ADSCs or BMSCs (Wang Y. et al., 2012), with acellular nerve grafts to repair nerve injury. Other researchers also focused on the application of cells in repairing peripheral nerve. In an interesting work, researchers found that gingiva-derived MSC-extracellular vesicles could activate the repair phenotype of Schwann cells and thereby promote nerve regeneration (Mao et al., 2018). In another work, conduits with spatially neurotrophic factors improved peripheral nerve repair (Sun A. X. et al., 2019). Liu’s group found that the acellular nerve allografts loaded with etifoxine or platelet-rich plasma can achieve a similar effect with autografts for nerve regeneration and functional recovery (Zheng C. et al., 2014; Zhou X. et al., 2014). Furthermore, Liu’s group also attempted to use these nerve grafts in clinic. The results of clinical trial from 72 patients showed that the human acellular nerve grafts could repair digital nerve injury in human (1–5 cm length) (He et al., 2015). They also used this human acellular nerve allograft to repair the injured nerves in the hand and upper extremity of patients (Zhu et al., 2017).

### Tendon/Ligaments

Tissue engineering strategy is also being used to treat tendon/ligament injury. Ouyang’s group has prepared nano-microfibrous PLGA or collagen/silk scaffold (Ouyang et al., 2003; Chen et al., 2008; Chen J. L. et al., 2010), or supplemented different cells, including MSCs (Ouyang et al., 2005), human embryonic stem cells (Chen et al., 2009), and human embryonic stem cell-derived MSCs (Chen J. L. et al., 2010) for tendon healing. Later, they found that the combination of histone deacetylase with well-aligned PLLA nanofibers may be more efficient to promote tenogenic differentiation of stem cells (Zhang et al., 2018a). Recently, they also attempted to use histone deacetylase inhibitor-treated tendon stem/progenitor cell-derived cell sheet to promote tendon repair (Zhang et al., 2018b). In another report, they modified the tendon ECM into a random-aligned-random composite, and the results showed that the biomimetic tendon ECM composite enhanced the bone and fibrocartilage formation in the interface (Liu H. H. et al., 2017). It was also reported that modifying the PLGA scaffold with fibronectin and type I collagen can promote the adhesion of human embryonic tenocytes (Qin et al., 2005). Xie’s group demonstrated that tissue-engineered tendons combined with cells and scaffolds could enhance the repair result (Yang et al., 2000). Cao and Liu’s groups used PGA unwoven fibrous scaffold seeded autologous dermal fibroblasts and tenocytes to repair Achilles tendon defect in a rabbit model (Deng et al., 2014). Later, they found that aligned nanofibers could facilitate *in vitro* tenogenic differentiation and *in vivo* tendon regeneration (Wang et al., 2016). They also found that tendon-like tissue was generated in a parthenogenetic stem cell-derived tenocytes-PLGA scaffold after *in vivo* heterotopic implantation (Liu W. et al., 2017). Recently, Lin’s group developed an oxidized HA/adipic acid dihydrazide hydrogel, the regimen of which could be used as a drug carrier and a mitigating agent of symptoms (Hsiao et al., 2019). This study provided a platform for exploring new therapeutics against tendinopathy.

### Cardiovascular Tissues

Cardiac disease remains one of the leading causes of death worldwide (Investigators et al., 1991). The goal of cardiovascular tissue engineering is to construct arteries, heart valves, and myocardium substitutes (Rabkin and Schoen, 2002). Similar to other fields of TERM, cardiovascular tissue engineering also consists of scaffolds, cells, and soluble mediators. Wang’s group constructed cardiac tissue, which could beat synchronously and similarly to native cardiac muscle by mixing embryonic stem cell-derived cardiomyocytes with collagen I (Guo et al., 2006). They also tried to use an electrically conductive double-network hydrogel consisting of poly(thiophene-3-acetic acid) and methacrylated aminated gelatin for cardiac tissue engineering (Yang et al., 2016). The injectable HA hydrogel, fullerenol/alginate hydrogel, and oligo(poly(ethylene glycol)fumarate) hydrogel, which could deliver stem cells or some therapeutic drugs, helped the treatment of myocardial infarction and were also developed (Lü et al., 2009; Wang H. et al., 2012; Hao et al., 2017). Ge’s group explored bioabsorbable sirolimus-loaded PLLA stents (Xinsorb) for coronary angioplasty. The preclinical and clinical outcomes of Xinsorb were further studied (Shen et al., 2017; Lv et al., 2018; Wu Y. Z. et al., 2019). Recently, they reported 5 years of serial intravascular imaging outcomes of Xinsorb scaffold (Wu Y. Z. et al., 2019). Other biomimetic vascular substitutes were also developed. Kong and Zhao’s groups developed many small-diameter vascular grafts based on PCL electrospun fibers (Wang Z. H. et al., 2015, 2017; Wang K. et al., 2017; Gong et al., 2016; Dong et al., 2018), for example, grafted fusion protein VEGF-HGFI on PCL to enhance vascular regeneration (Wang K. et al., 2017). A bilayered vascular graft aiming to improve hemocompatibility and endothelial cell monolayer formation was also constructed using smooth PCL surface as internal layer and PCL microfibers as external layer (Dong et al., 2018). In a recent work, they found that MSC-derived small extracellular vesicles functionalized PCL electrospun grafts could enhance patency and inhibit calcification by immunomodulation in a rat model of hyperlipidemia (Wei et al., 2019). Han’s group reported that a dual functionalized vascular stent prepared by spatio-temporal coating of HA conjugated with dopamine showed improved neointimal area and inflammation score in a porcine restenosis model (Kim et al., 2016). 3D printing is also a promising method to construct biomimetic vasculature for tissue regeneration. For instance, Lei et al. (2019) developed perfusable and hierarchical networks with microchannels (PHMs) via the combination of 3D-printed sacrificial caramel templates, polymer coating, and phase separation. The PHMs could support the metabolic functions of heart cells *in vitro* and efficiently treat myocardial infarction.

**FIGURE 2:**
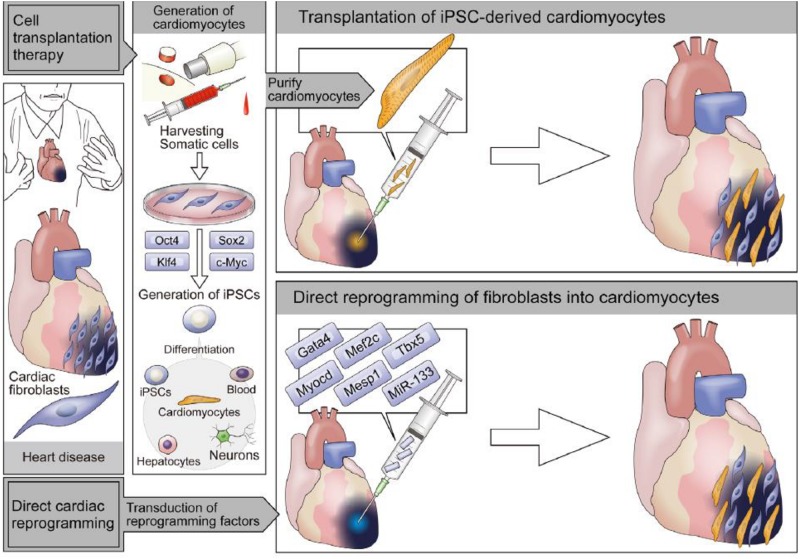
Future cardiac regenerative therapy by cell transplantation-based approach using iPS cell-derived cardiomyocytes **(Upper)** and direct cardiac reprogramming approach **(Lower)** (reproduced with permission from Sadahiro et al., 2015).

Current heart transplantation therapy is still limited by lacking donors, suffering from chronic immunosuppressant therapy, and taking risks of subsequent development of graft vasculopathy (Stehlik et al., 2011). TERM provides several potential strategies for heart regeneration. The mammalian myocardium of human only has a limited regenerative capability. *In situ* tissue regeneration through mobilizing endogenous cell has been widely investigated. In a study, the phosphorylated form of 7A was loaded in collagen hydrogel to promote regeneration of infarcted myocardium and improve heart function in a rodent model (Zhang Y. et al., 2019). Decellularized matrix is also used for heart regeneration. Dai et al. (2019) fabricated a composite scaffold by combining a porous matrix metalloproteinase degradable PEG hydrogel with SDF-1α. The findings suggested that the SDF-1α-loaded hydrogel was efficient to develop functional decellularized heart valve. MicroRNA was also used to promote cardiac regeneration. For example, Chen’s group proved that miR-19a/19b had a therapeutic effect in cardiac regeneration and protection (Gao et al., 2019). In addition, Sun’s group and Wang’s group successfully generated human ventricular-specific heart tissue (EhVHT) based on 3D Fe oxide scaffolds (Yang H. et al., 2019). This EhVHT could not only meet the specific requirements for ventricular damages in most myocardial infarction but also be used to screen drugs targeting ventricular myocardium. Another way of TERM is to induce differentiation of cardiac progenitor cells into cardiomyocytes in damaged hearts (Garbern and Lee, 2013; Senyo et al., 2013). Exogenous sources, such as embryonic stem cells and iPSCs, are also used to repair the heart (Shiba et al., 2012). In a study, mouse cardiac fibroblasts (CFs) were reprogrammed into induced cardiomyocyte-like cells (iCMs) in the presence of Mef2c, Gata4, and Tbx5 (Ieda et al., 2010). Human fibroblasts were also reported to be induced into cardiomyocyte-like cells (Fu et al., 2013; Wada et al., 2013). To improve the survival of transplanted cells and reprogramming efficiency of iPSCs in the injured heart, *in vivo* reprogramming could generate more mature iCMs. Therefore, directly injecting the reprogramming factors into the damaged heart may convert the endogenous CF population into new functional iCMs. Compared with *in vitro* reprogramming method, this *in vivo* reprogramming approach, which is simple, has lower risk of tumor formation, and avoids cell transplantation, may be a future cardiac regenerative therapy (Figure 2; Sadahiro et al., 2015). With this method, the diseased myocardial tissue covered by fibrous tissue consisting of CFs and ECM can be converted into cardiomyocytes by defined factors *in situ* by direct cardiac reprogramming approach using defined factors.

### Dental Tissues

A vast array of metals including gold, silver, Cu, palladium, platinum, nickel, and Ti have been widely used for dental restorations. However, the oral environment will cause corrosion of metallic restorations and generation of metal ions. Ti is a common material used in dentistry. In particular, the modulus of Ti alloys could be regulated similarly to human cortical bone by Yang’s group to reduce the stress-shielding effect after implantation (Hao et al., 2007a, b). However, the surface of Ti alloys is basically bioinert, which means it is not conducive to osseointegration. Huang’s group attempted to use nano/submicron-scale TiO_2_ network (Yang et al., 2009), oxygen plasma immersion ion implantation treatment (Yang C. H. et al., 2015), and genipin (Sun Y. S. et al., 2018) for improving cell response on Ti surface. Recently, they developed a multiform nano-network of TiO_2_ on Ti surface. This multiform nanostructure was shown to enhance proliferation and differentiation of human BMSCs and regulate the formation of focal adhesion complex (Yang and Huang, 2019). Hwang’s group found that UV treatment of large grit sand-blasting and acid-etching implants followed by wet storage could enhance bioactivity (Choi et al., 2019). Different from other methods using bone matrix components to promote cell adhesion on the surface of implant, Wang’s group developed a polysaccharide coating that could improve osseointegration by regulating macrophages into a pro-regenerative phenotype and producing abundant osteogenic/angiogenic cytokines to enhance osteogenesis locally (Shi et al., 2018). Vijayalakshmi’s group fabricated Ag HAp/functionalized multiwall carbon nanotube (Ag-HA/f-MWCNT) T coating on passivated 316L stainless steel as a novel alternative to dental implant. The results showed that 3 wt% Ag-substituted HA/f-MWCN coating is nonhemolytic and most suited (Sivaraj and Vijayalakshmi, 2019). Cho and Kim’s groups found that 3D-printed cobalt–chrome (Co–Cr) alloys were more biocompatible than nickel–chrome (Ni–Cr) alloys (Ganbold et al., 2019).

In addition, other materials and their functions were also determined. Tsuji group reported a fully functional tooth replacement in mouse through the transplantation of bioengineered tooth germ into the alveolar bone (Ikeda et al., 2009). For the purpose of better esthetics, zirconia ceramics may be an alternative to Ti (Miyazaki et al., 2013). The zirconia ceramics could be fabricated into fixed dental prostheses, by computer-aided/computer-aided manufacturing system. Nakamura et al.’s (2019) group evaluated the mechanical properties of some zirconia-based materials (Itakura et al., 2019). Lee group proved that zirconia implants with acid-etched showed comparable osseointegration with Ti implants (Kuo et al., 2017). Triethylene glycol dimethacrylate (TEGDMA) and mineral trioxide aggregate (MTA) are also common materials used in dentistry. Peng’s group found that the autophagy was activated by TEGDMA via the AMPK/mTOR pathway, but this effect could be abrogated by *N*-acetyl cysteine pretreatment (Zhu et al., 2015). Shie group evaluated the feasibility of MTA powder coated with PDA and MTA/PCL scaffold by 3D printing in dental tissue regeneration, and found that these materials showed potential application in dental tissue engineering (Chiu et al., 2017; Tu et al., 2018). Lim group found that modified MTA with propolis could promote odontogenic differentiation and mineralization of human dentalpulp stem cells (DPSCs) through ERK pathway (Kim J. H. et al., 2019). However, Lee’s group thought that it should be careful consideration of the concentration of MTA extracts, especially when applying MTA to the elderly patients to maintain the viability of human MSC (Kim S. Y. et al., 2019). Shie group prepared mesoporous calcium silicate nanoparticles as endodontic materials, which could have osteogenic, drug delivery, and antibacterial characteristics (Huang et al., 2017). iRoot BP Plus, a promising MTA alternative, were proved to promote *in vitro* recruitment of DPSCs and facilitate dentin bridge formation in a pulp repair model *in vivo* (Zhu et al., 2014). Honda group prepared protamine-loaded dicalcium phosphate anhydride (DCPA), which showed excellent antimicrobial activity against *Streptococcus mutans* (Fujiki et al., 2019). Lee and Kim’s groups developed a Sr ion-releasing nanobiocement (Sr-NBC) based on sol–gel method. This Sr-NBC showed excellent biocompatibility and high odontogenic potential *in vitro* and more new dentin formation *in vivo* (Mandakhbayar et al., 2019). Anastasiou group incorporated Sr^2+^ or Ce^3+^ ions doped fluorapatites into chitosan scaffold, the results displayed that Sr^2+^ presented high osteoconductivity while Ce^3+^ could retain the good antibacterial property and osteoconductivity (Anastasiou et al., 2019). Lee group used chlorhexidine has been incorporated into the composition of PMMA dental restorations to enhance their antimicrobial performance (Mai et al., 2019). Bindal et al.’s (2019) group determined that 20% human platelet lysate could be optimum for maintaining the *in vitro* proliferative and angiogenic potential of inflamed dental pulp stem cells. Venkatasubbu group developed polyvinyl alcohol/alginate/HAp films loaded with amoxicillin, and the loading amoxicillin helped in healing the infection while HAP nanoparticles helps in periodontal regeneration (Prakash et al., 2019).

## Concluding Remarks

**FIGURE 3:**
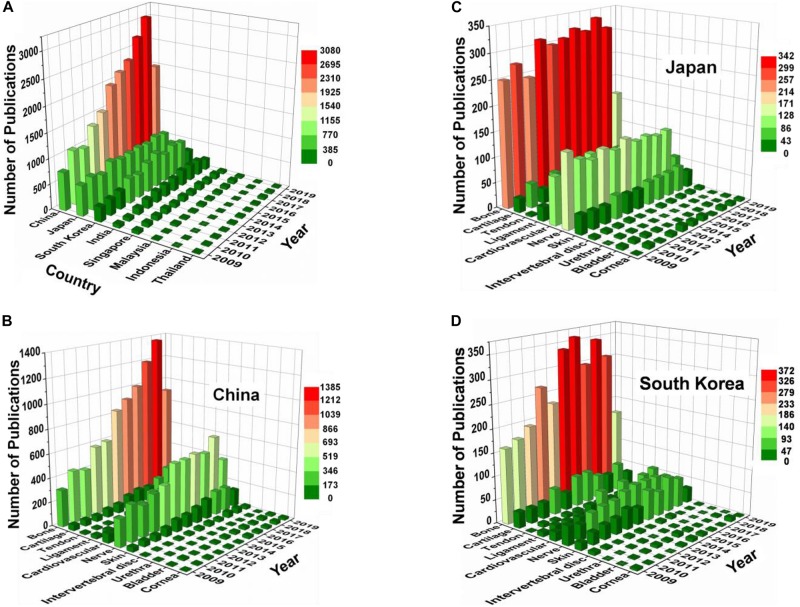
The number of publications in different Asian countries **(A)**, and different fields in China **(B)**, Japan **(C)**, and South Korea **(D)** from 2009 to 2019.

We used the Wed Web of Science database to search for original articles on the topics of “Bone,” “cartilage,” “tendon/ligament,” “cardiovascular,” “nerve,” “skin,” “intervertebral disc,” “urethra,” “bladder,” “cornea,” and “regeneration” published between 2009 and August 2019 inclusively. In Asia, China is one of the leading countries in regenerative medicine research, as shown in Figure 3. Based on the quantity of publications, China, Japan, South Korea, India, and Singapore have paid more attention on TERM.

First of all, we must admit that this review of recent advances in TERM in Asia is far from comprehensive and a large number of excellent research teams and their work might be missed due to limited space. To date, great progress in TERM has been made by Asian researchers, not only by their publications, but also by their active efforts to promote the clinical transformation of their research results. We believe that TERM will experience a rapid development, because Asian governments and society are increasingly concerned with TERM. For example, in China, regenerative medicine is regarded as one of the five biotechnology fields in the National Medium to Long-term Plan for Scientific and Technological Development (2006–2020). There are also many foundations to support the basic research and clinical transformation of TERM.

Biomaterials, various cell sources, and bioactive factors are essential to TERM. Asian scholars have studied polymer, ceramic, and metal for TERM with the aim of having different applications. There are various preparation methods to construct biomaterials-based scaffold. Among them, 3D bioprinting and microfluidics technology excel due to their precision and controllability in preparing personalized materials and complex tissues/organs. In particular, cell sheet is a novel technique first proposed by Asian scholars. Except for the preparation method, the development in chemistry and materials also promotes the rapid development of biomaterials-based scaffold. Through the chemistry modification, we could improve the properties of the existing materials. A variety of new materials are also created and used in TERM. Furthermore, the materials could be processed into hydrogels, porous scaffolds, and fibers, to meet the different needs of TERM. In the future, researchers will continue to develop numerous functional biomaterials to engineer tissue-specific scaffolds.

The extension of cell sources also promotes the development of TERM. To date, the seed cells are no longer confined to autologous adult cells. Various multipotent cells, including embryonic stem cells, bone marrow stem cells, tissue-specific progenitor stem cells, umbilical cord stem cells, and iPS cells, are proved to be effective in TERM. In particular, the iPSC cell is a novel discovery by Asian scholars. Furthermore, the generation of iPS cell lines by Asian scholars, which could generate fertile progeny by tetraploid complementation, is also a breakthrough. In addition, the direct *in vivo* reprogramming approach, which is reported simple, has a lower risk of tumor formation and avoids cell transplantation as proposed by Asian scholars. The future direction of stem cells is how to induce stem cell into specific cells showing similar functions with adult cells (Tabar and Studer, 2014). It is predictable that the future of TERM may be updated with stem cell technologies (Tang et al., 2017). Here, we provide some of the recent preclinical and clinical studies using stem cell therapy. In the clinic, allogeneic MSCs from bone marrow or umbilical cord have been evaluated in cartilage defect, SCI, corneal injuries, cardiomyopathy, IVD degeneration, and bone defect with an excellent safety profile. The available clinical studies in cell transplantation indicate that some autologous and allogeneic stem cell therapy from different sources is safe (Table 3).

**TABLE 3:** Examples of clinical trials on MSC cell therapy.

NCT	Disease	MSC source	Objectives	Country	Status
NCT01302015	Buerger’s disease	Autologous ADSCs	Treat patient with Buerger’s disease	South Korea	Completed Estimated study Completion Date: June 2019
NCT01309061	Romberg’s disease	Human ADSCs	Treat patient with progressive hemifacial atrophy	South Korea	Completed Estimated study Completion Date: June 2019
NCT00979758	Myocardial infarction	Bone marrow mononuclear cells	Improve the outcome of patients with impaired left ventricle function after myocardial infarction	China	Completed Estimated study Completion Date: January 2018
NCT02948023	Corneal injuries	*Ex vivo* cultivated limbal stem cells	Treat different superficial corneal pathologies in human	India	Recruiting Estimated study Completion Date: March 2018
NCT02260713	Acute spinal cord injury	Autologous bone marrow cells	Treat acute complete spinal cord injury	India	Completed Estimated study Completion Date: March 2018
NCT02669199	Large area skin wound injury	Allogeneic umbilical cord derived MSCs	Treat large area skin lesions of the subjects	China	Recruiting Estimated study Completion Date: February 2016
NCT00981006	Ischemic cardiomyopathy	Autologous human cardiac-derived stem cell	Treat severe refractory heart failure patients with chronic ischemic cardiomyopathy concordance with reduced left ventricular dysfunction	Japan	Completed Estimated study Completion Date: April 2015
NCT02338271	Intervertebral disc degeneration	Autologous ADSCs	Treat chronic low back pain patients with lumbar intervertebral disc degeneration	South Korea	Completed Estimated study Completion Date: January 2015

In order to promote the development of TERM, it is also important to better understand and replicate the microenvironment around tissue/organ. The improvement of microenvironment may be realized by applying bioactive factors, including growth factors, chemokines, cytokines, or other signals, such as electric, mechanical, and magnetic stimulation.

The ultimate goal of TERM is to translate the findings of basic research into the clinic. The mineralized collagen, which mimics the natural bone ECM, has acquired the product registration certificate from China government. Some nerve scaffolds, including human acellular nerve grafts and chitosan-based grafts, have been promoted the clinical translation in China (Fan et al., 2008; Gu et al., 2012; Hvistendahl, 2012; He et al., 2015; Zhu et al., 2017). Using a composite of allogeneic umbilical cord blood-derived MSCs and hyaluronate hydrogel, regenerated articular cartilage in the defects of patients was observed (Park Y. B. et al., 2017). Bioabsorbable sirolimus-loaded PLLA stents (Xinsorb) as an alternative to metallic stents for coronary angioplasty showed 5 years of serial intravascular imaging in patients (Wu Y. Z. et al., 2019). There have been many commercial technologies and products such as 3D printing. Two well-known 3D bioprinters, “Life-Printer ‘X”’ and “CellJet Cell Printer,” are developed in Singapore and Japan, respectively (Arslan-Yildiz et al., 2016). Several examples of 3D bioprinting companies and their products and projects are shown in Table 4. In a review on 3D-printing clinical trials for the world, 3D-printing trials were registered in 20 different countries, most commonly in China with 42 (45.65%) trials (Witowski et al., 2018). The application of cell sheet in the clinic includes heart, cornea, blood vessels, periodontal membrane, esophagus, cartilage, functional tendons, and middle ear (Nishida et al., 2004; Egami et al., 2014; Iwata et al., 2015; Yamamoto et al., 2017). Okano’s group has done some clinical trials in humans for treating SCI using iPSC-NPCs (Isoda et al., 2016; Nagoshi and Okano, 2018; Tsuji et al., 2019). In the past decades, part of the tissue-engineered products has been applied to the clinic (Tang et al., 2017). However, they make up only a very small percentage. Although the therapeutic effect of cell–materials composite is better than materials, most successful products are still biomaterial-based scaffolds. The clinical application of stem cells is mostly involved in hematological diseases. In the future, we still have a long way to go in combining TERM with the clinic.

**TABLE 4:** Examples of 3D bioprinting companies and products in Asia.

Company	Project/products	Founding year	Country	Website
Next 21	Custom-made artificial bone implants	2000	Japan	http://next21.info
Osteopore International	Osteoplug; Osteomesh	2003	Singapore	http://osteopore.com.sg
Cyfuse Biomedical	Bone, cartilage, blood vessel, neural tissue, liver	2010	Japan	http://cyfusebio.com
Regenovo Biotechnology	Bone, blood vessel, cartilage	2013	China	http://regenovo.com
SUNP BIOTECH	Custom-made products, bioink, bioprinter	2014	China	http://sunpbiotech.com
Rokit	Skin, bioink, bioprinter		South Korea	https://onsbio.com/bioprinting
Pandorum Technologies	Liver, cornea	2011	India	http://www.pandorumtechnologies.com

## Author Contributions

BL and FH conceived and designed the review. FH wrote the manuscript. JW, LD, YH, ZY, QG, CZ, LY, HW, ZZ, LJ, JL, YY, WZ, and GC all participated in the data search and analysis. WL and SC assisted with the revision of this manuscript.

## Conflict of Interest

The authors declare that the research was conducted in the absence of any commercial or financial relationships that could be construed as a potential conflict of interest.
